# Hydrolytic Enzymes as Potentiators of Antimicrobials against an Inter-Kingdom Biofilm Model

**DOI:** 10.1128/spectrum.02589-21

**Published:** 2022-02-23

**Authors:** Albert Ruiz-Sorribas, Hervé Poilvache, Nur Hidayatul Nazirah Kamarudin, Annabel Braem, Françoise Van Bambeke

**Affiliations:** a Pharmacologie Cellulaire et Moléculaire, Louvain Drug Research Institute, Université catholique de Louvain, Brussels, Belgium; b Laboratoire de neuro musculo squelettique, Institut de Recherche Expérimentale et Clinique, Université catholique de Louvain, Brussels, Belgium; c Orthopaedic Surgery Department, Cliniques Universitaires Saint-Luc, Brussels, Belgium; d Department of Materials Engineering, Biomaterials and Tissue Engineering Research Group, KU Leuven, Leuven, Belgium; e Department of Chemical and Process Engineering, Faculty of Engineering and Built Environment, Universiti Kebangsaan Malaysia, Bangi, Malaysia; University of Debrecen

**Keywords:** biofilms, *S. aureus*, *E. coli*, *C. albicans*, enzymes, antimicrobials

## Abstract

Biofilms are recalcitrant to antimicrobials, partly due to the barrier effect of their matrix. The use of hydrolytic enzymes capable to degrade matrix constituents has been proposed as an alternative strategy against biofilm-related infections. This study aimed to determine whether hydrolytic enzymes could potentiate the activity of antimicrobials against hard-to-treat interkingdom biofilms comprising two bacteria and one fungus. We studied the activity of a series of enzymes alone or in combination, followed or not by antimicrobial treatment, against single-, dual- or three-species biofilms of Staphylococcus aureus, Escherichia coli, and Candida albicans, by measuring their residual biomass or culturable cells. Two hydrolytic enzymes, subtilisin A and lyticase, were identified as the most effective to reduce the biomass of C. albicans biofilm. When targeting interkingdom biofilms, subtilisin A alone was the most effective enzyme to reduce biomass of all biofilms, followed by lyticase combined with an enzymatic cocktail composed of cellulase, denarase, and dispersin B that proved previously active against bacterial biofilms. The subsequent incubation with antimicrobials further reduced the biomass. Enzymes alone did not reduce culturable cells in most cases and did not interfere with the cidal effects of antimicrobials. Therefore, this work highlights the potential interest of pre-exposing interkingdom biofilms to hydrolytic enzymes to reduce their biomass besides the number of culturable cells, which was not achieved when using antimicrobials alone.

**IMPORTANCE** Biofilms are recalcitrant to antimicrobial treatments. This problem is even more critical when dealing with polymicrobial, interkingdom biofilms, including both bacteria and fungi, as these microorganisms cooperate to strengthen the biofilm and produce a complex matrix. Here, we demonstrate that the protease subtilisin A used alone, or a cocktail containing lyticase, cellulase, denarase, and dispersin B markedly reduce the biomass of interkingdom biofilms and cooperate with antimicrobials to act upon these recalcitrant forms of infection. This work may open perspectives for the development of novel adjuvant therapies against biofilm-related infections.

## INTRODUCTION

Biofilms are consortia of microorganisms embedded in an auto- or host-produced matrix and attached or not to biotic or abiotic surfaces (1). Medical devices are particularly prone to colonization by biofilms, which are found in infections developing on vascular or urinary catheters, cardiac valves, urethral stents, endotracheal tubes, joint prostheses, etc (1, 2). The treatment of these infections usually requires additional surgery, with exhaustive washing of the site of infection, local and/or general antibiotherapies, replacement of the medical device and long periods of invalidity for the patients (3, 4). Nevertheless, treatment failure is frequent, leading to chronic or relapsing infections, bloodstream infection and, in the most severe cases, death (5). In this context, polymicrobial infections, in which at least two different pathogens are identified on the same site of infection, and, more particularly, interkingdom biofilms, with at least a bacterium and a fungus, are more recalcitrant and require longer treatments (6–9).

Biofilms are refractory to antibiotics and immune defenses for several reasons. First, the matrix, composed of extracellular polymeric substances (EPS) as extracellular DNA, proteins and polysaccharides, is a barrier that reduces the diffusion and bioavailability of drugs (10) as well as the access of immune cells (11, 12). Second, the scarcity of nutrients and oxygen in the biofilm triggers the appearance of dormant phenotypes. These cells with low metabolic levels are not responsive to antibiotics requiring an active metabolism and replication to exert their antimicrobial effects (13). Lastly, dormant microorganisms surviving antibiotics may regain a functional metabolism and act as a reservoir explaining relapses of the infection (14).

Alternative strategies aiming at acting on dormant microbes or at destabilizing the matrix are under investigation to try eradicating biofilms in the context of medical device-associated infections. They include, among others, antimicrobial peptides, metallic nanoparticles, bacteriophages, or hydrolytic enzymes (15–19). The use of hydrolytic enzymes is a strategy that mimics the natural process of biofilm dispersal and aims to the degradation of EPS composing the matrix (20, 21). The hydrolytic activity of nucleases, lysins, peptidases and glycoside hydrolases has been previously reported (22–25). Consequently, the use of formulations of hydrolytic enzymes in a clinical context is of high interest, especially when combined with antimicrobial therapy to avoid a sudden release of dispersed microbes in the host (26). Given the diverse chemical nature of EPS found in biofilm matrix, combining several enzymes can broaden their spectrum of activity. A previously described formulation including cellulase (Ce), denarase (De) and dispersin B (Di), formerly referred to as TEC (Three Enzymatic Cocktail), proved highly effective to disrupt biofilms formed by common bacterial pathogens like Staphylococcus aureus, Pseudomonas aeruginosa, Escherichia coli, or Klebsiella pneumoniae and to reduce the culturable cells in the biofilms when combined with antibiotics (27–29). Cellulase and dispersin B are endoglycosidases targeting with the highest affinity the β(1–4)-O-glycosidic linkages (30) and poly-β(1–6)-*N-acetylglucosamine* (31, 32) that are abundant in biofilms from these species. Denarase is a genetically engineered endonuclease with DNase and RNase activity (33). This cocktail has not yet been tested against multispecies biofilms, especially those comprising also a fungus, despite their clinical importance.

This work explores the use of hydrolytic enzymes as potentiators of antimicrobials against interkingdom biofilms, with the specific aim to enlarge the activity of the previously described cocktail of enzymes to fungal biofilms. To this effect, a panel of hydrolytic enzymes was screened against C. albicans biofilms and the activity of the selected enzymes was analyzed in combination with a tri-enzymatic cocktail of Ce/De/Di and antimicrobials against a recently published interkingdom three-species biofilm model (34). This model includes S. aureus as the opportunistic pathogen with the highest prevalence in medical devices associated infections, E. coli as a model for Enterobacteriaceae infections, and C. albicans as the most frequently isolated fungus (35, 36).

## RESULTS

### Screening of hydrolytic enzymes against C. albicans biofilms.

**FIG 1:**
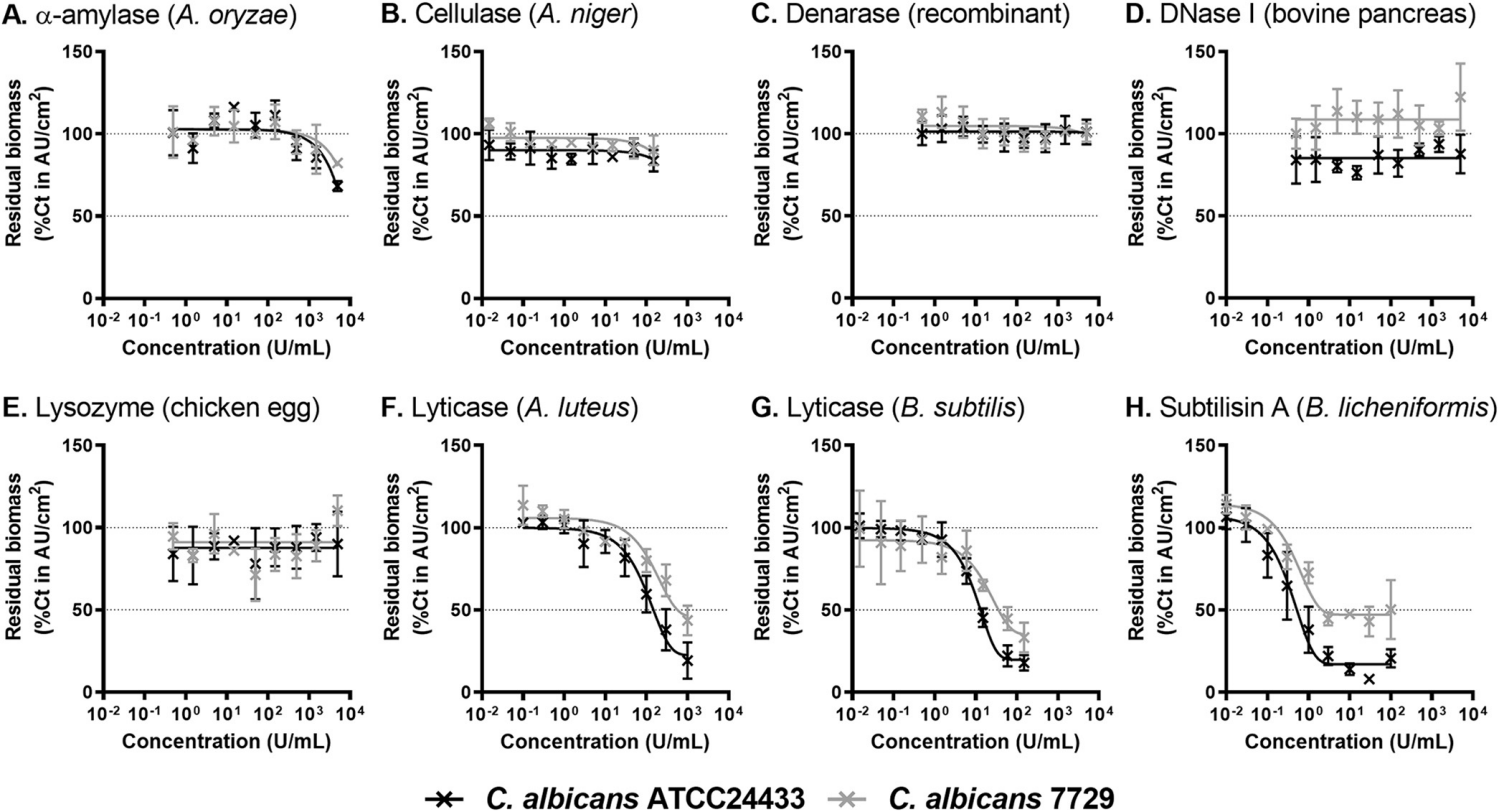
Activity of hydrolytic enzymes to reduce biomass of C. albicans ATCC 24433 (black) and C. albicans 7729 (gray) biofilms. The graphs show the reduction in crystal violet absorbance, in percentage of control values (no enzyme added) measured after 1 h of incubation with enzymes over a broad range of concentrations. A: alpha-amylase; B: cellulase; C: denarase; D: DNAse I; E: lysozyme; F: lyticase from A. luteus; G: lyticase from B. subtilis; H: subtilisin A. Data are mean ± SD of 3 independent experiments.

A panel of selected hydrolytic enzymes were screened for their capacity to reduce the biomass of C. albicans biofilms formed by a reference and a clinical strain. α-amylase, cellulase, denarase, DNase I and lysozyme (Fig. 1A–E) were poorly or not active over the range of concentrations tested. Lyticase from *Arthrobacter luteus* and Bacillus subtilis (Fig. 1F and G) showed a concentration-dependent activity, with IC_50_ of 162.2 and 12.6 U/mL against the reference strain, and 812.8 and 39.4 U/mL against the clinical strain, respectively. Subtilisin A from Bacillus licheniformis (Fig. 1H) was the most potent enzyme with IC_50_ of 0.5 and 5.18 U/mL against the reference and clinical strains, respectively. The lyticase from B. subtilis and the subtilisin A from B. licheniformis were selected for further experiments as they were the most potent against C. albicans biofilms. In addition, the kinetics of reduction of biomass in C. albicans biofilm by these two enzymes was tested (Fig. S1). The enzymatic activity progressed overtime against the ATCC 24433 strain while a plateau of maximal efficacy was reached for incubation of 30 (subtilisin A) to 60 (lyticase) minutes against the clinical isolate 7729. Moreover, the maximal activity of the tri-enzymatic cocktail Ce/De/Di was previously described at 30 min (28). Thus, a time point of 60 min was selected for further experiments as the shortest time where all enzymes showed the maximal effect.

### Hemolysis and cytotoxicity of the hydrolytic enzymes.

**FIG 2:**
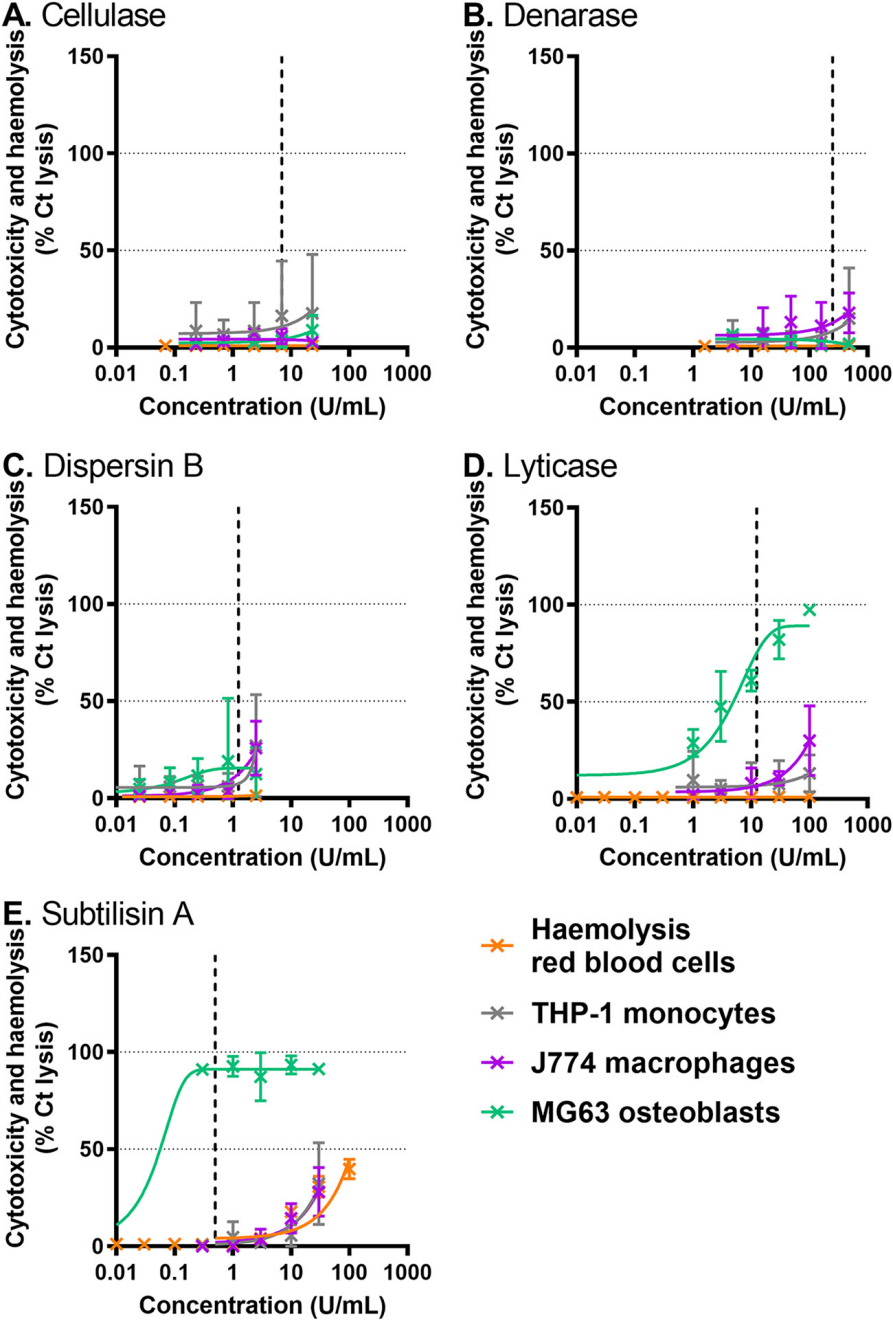
Cytotoxicity and hemolysis of hydrolytic enzymes against THP-1 monocytes (gray), J774 macrophages (purple), MG63 osteoblasts (vert) and red blood cells (orange). The graphs show the percentage of cytotoxicity or hemolysis recorded after 1 h of incubation with enzymes at the indicated concentrations (100%: triton 1%). A: cellulase; B: denarase; C: dispersin B; D: lyticase from B. subtilis; E: subtilisin A. Vertical dotted line: concentration used in following experiments. Data are mean ± SD of 3 independent experiments.

The hemolytic activity and the cytotoxicity of the hydrolytic enzymes active against C. albicans (lyticase and subtilisin A), together with that of cellulase, denarase and dispersin B were tested (Fig. 2).

Cellulase, denarase and dispersin B showed no or very low hemolytic activity and cytotoxicity over the range of concentrations tested (Fig. 2A–C). Lyticase and subtilisin A caused cytotoxicity against monocytes or macrophages at the highest concentrations tested and subtilisin A also induced hemolysis (<50%) for concentrations higher than 10 U/mL (Fig. 2D and E). Importantly, both enzymes were highly cytotoxic against osteoblasts, causing, respectively 75.1 and 91.2% reduction in their metabolic activity at the concentrations used in further experiments.

### Incubation with hydrolytic enzymes.

**FIG 3:**
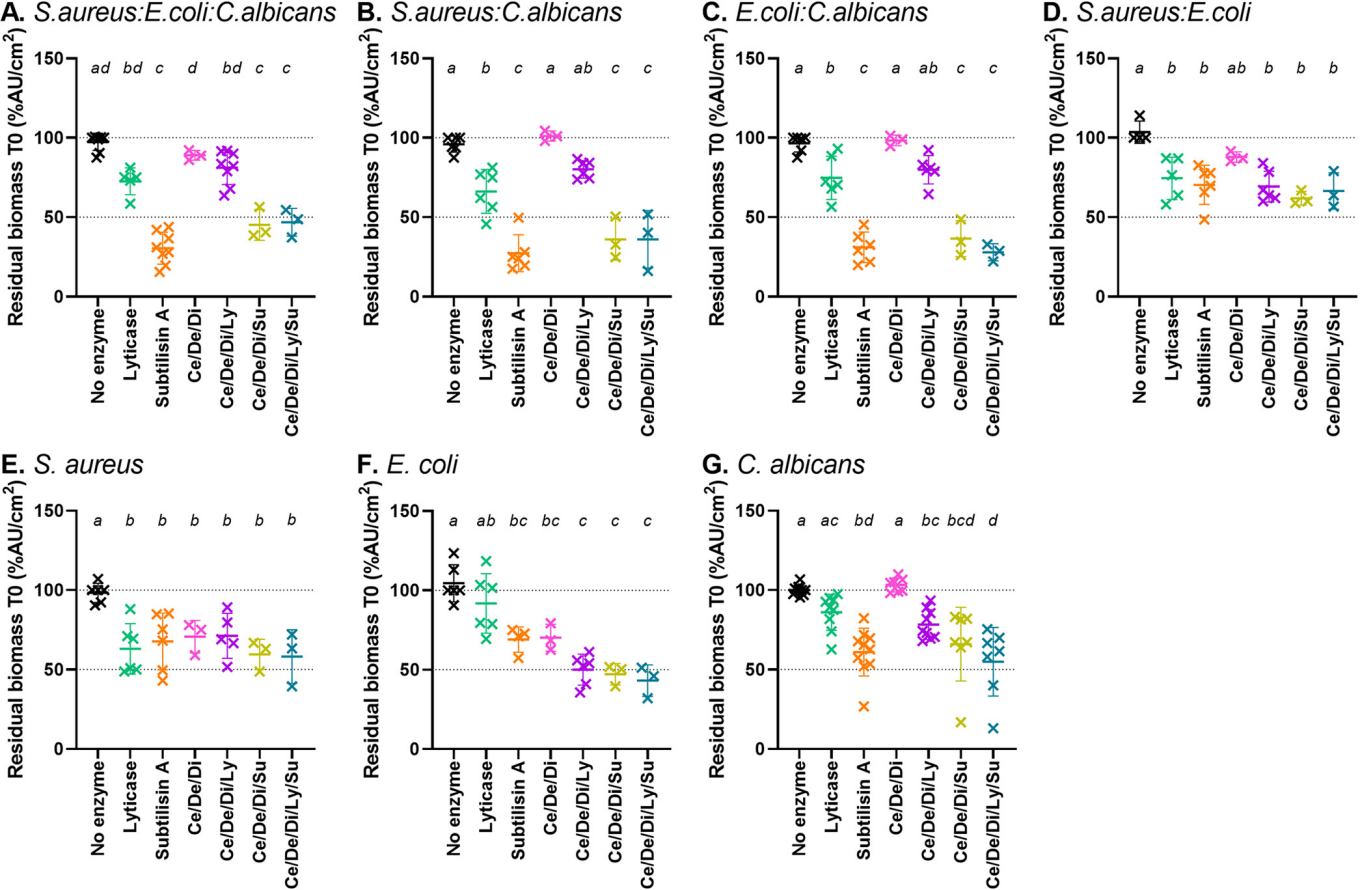
Biomass reduction of biofilms after 1 h of incubation with hydrolytic enzymes (corresponding to T0 in further experiments). Each panel corresponds to a specific biofilm model (A: three-species; B-D: dual species with the indicated species; E-G: single species). Enzyme’s concentrations were 7 U/mL for cellulase (Ce), 250 U/mL for denarase (De), 1.25 U/mL for dispersin B (Di), 12.6 U/mL for lyticase (Ly), and 0.5 U/mL for subtilisin A (Su). Symbols represent the mean of replicates from each of 3 to 10 independent experiments. Horizontal bars represent the mean ± SD of all values. Statistical analyses: conditions with different letters are significantly different (*P* < 0.05; One-way ANOVA, Tukey posttest).

The activity of lyticase, subtilisin A, and the Ce/De/Di cocktail was tested against the three-species *S.aureus:E.coli:C.albicans* biofilm and the subsequent dual- and single-species biofilms. From this point forward, lyticase and subtilisin A were used at the IC_50_ against the reference strain (12.6 and 0.5 U/mL, respectively). The results for biomass and culturable cells are represented in Fig. 3 and Fig. S2, respectively, and the ANOVA results are detailed in Table S1.

First, the Ce/De/Di cocktail significantly reduced the biomass of bacterial single-species biofilms (Fig. 3E and F) but not that of multispecies biofilms (Fig. 3A–D). Lyticase and subtilisin A alone caused significant decreases in biomass for all models, except for lyticase against single-species E. coli and C. albicans biofilms (Fig. 3F and G). The effect of subtilisin A was significantly larger than that of lyticase in biofilms where C. albicans was present (Fig. 3A–C and G). When lyticase was co-incubated with the Ce/De/Di cocktail, a significantly larger reduction in biomass was observed only against E. coli biofilm (Fig. 3F). On the other hand, the combination of subtilisin A with the Ce/De/Di cocktail, combined or not with lyticase, did not improve the activity compared to subtilisin A alone. None of the enzymes, alone or in combination, was able to reduce the numbers of culturable cells in all biofilm models (Fig. S2).

As our goal was to obtain the broadest spectrum of action possible, we considered a combination of lyticase with the Ce/De/Di cocktail as the formulation of choice for further experiments and compared it with subtilisin A as the most active enzyme when used alone.

### Sequential incubation with hydrolytic enzymes and antimicrobials.

**FIG 4:**
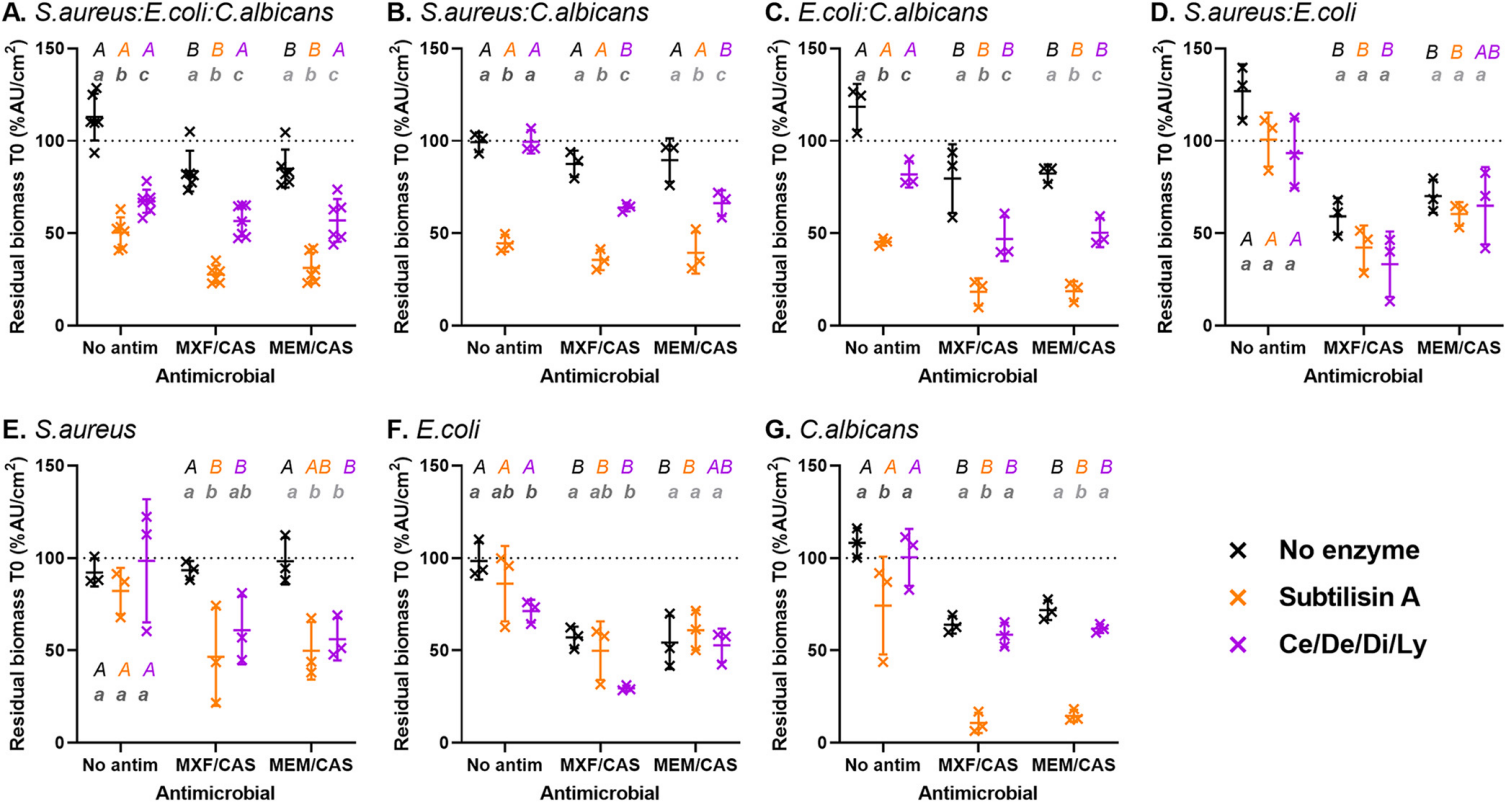
Biomass reduction of biofilms after sequential incubation with hydrolytic enzymes during 1 h and antimicrobial agents during 24 h (T24). Each panel corresponds to a specific biofilm model (A: three-species; B-D: dual species with the indicated species; E-G: single species). Data are expressed as the percentage of the value measured at the end of the preincubation with enzymes (T0). The enzymes tested are subtilisin A 0.5 U/mL (orange) or cellulase 7 U/mL/denarase 250 U/mL/dispersin B 1.25 U/mL/lyticase 12.6 U/mL (Ce/De/Di/Ly, purple) versus no enzyme (black), and antimicrobials, moxifloxacin 4 mg/L/caspofungin 13.8 mg/L (MXF/CAS) or meropenem 40 mg/L/caspofungin 13.8 mg/L (MEM/CAS). Symbols represent the mean of replicates from each of 3 to 6 independent experiments. Horizontal bars represent the mean ± SD of all values. Statistical analysis: different lowercase letters of the same grayscale color denote significant difference among enzymes for each antimicrobial incubation; different uppercase letters of the same color denote significant difference among antimicrobials for each enzymatic incubation (*P* < 0.05; Two-way ANOVA, Tukey posttest).

**FIG 5:**
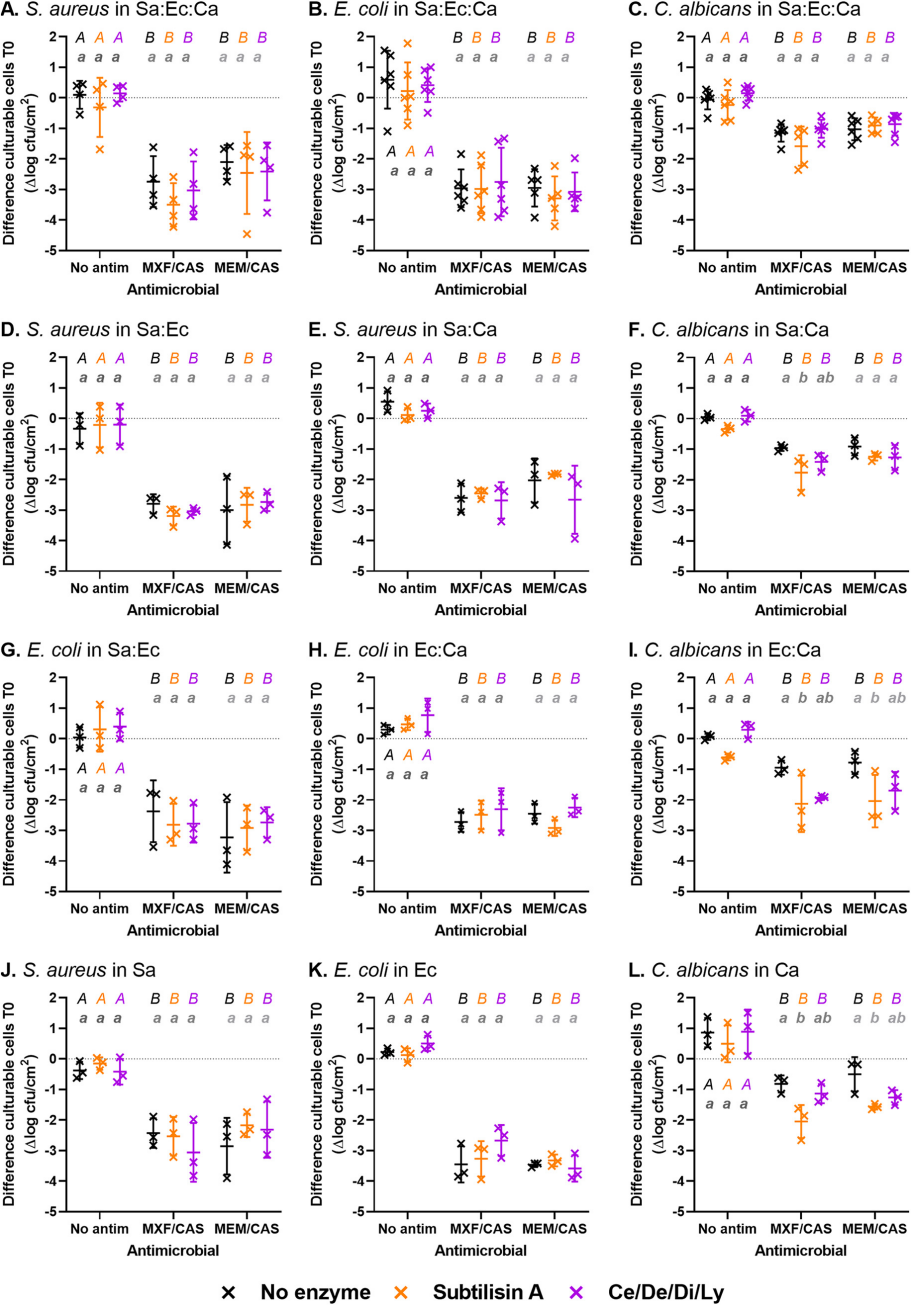
Culturable cells reduction of biofilms after sequential incubation with hydrolytic enzymes during 1 h and antimicrobial agents during 24 h (T24). Each panel shows the counts for one species (indicated in italics after the letter of the panel) in a specific biofilm model (A-C: three-species; B-I: dual species with the indicated species; J-L: single species; Sa: S. aureus; Ec: E. coli; Ca: C. albicans). Data are expressed as the percentage of the value measured at the end of the preincubation with enzymes (T0). The enzymes tested are subtilisin A 0.5 U/mL (orange) or cellulase 7 U/mL/denarase 250 U/mL/dispersin B 1.25 U/mL/lyticase 12.6 U/mL (Ce/De/Di/Ly, purple) versus no enzyme (black), and antimicrobials, moxifloxacin 4 mg/L/caspofungin 13.8 mg/L (MXF/CAS) or meropenem 40 mg/L/caspofungin 13.8 mg/L (MEM/CAS). Symbols represent the mean of replicates from each of 3 to 6 independent experiments. Horizontal bars represent the mean ± SD of all values. Statistical analysis: different lowercase letters of same grayscale color denote significant difference among enzymes for each antimicrobial incubation; different uppercase letters of the same color denote significant difference among antimicrobials for each enzymatic pre-incubation (*P* < 0.05; Two-way ANOVA, Tukey posttest).

The activity of the combination of hydrolytic enzymes and antimicrobials was tested by incubating first the biofilms with enzymes for 1 h (T0) and then adding the antimicrobials for 24 h (T24). This protocol was similar to that previously adopted to study the activity of the Ce/De/Di cocktail against single species bacterial biofilms (28) and aimed at mimicking a therapeutic approach in which the enzymes would be briefly applied in the vicinity of an infected implanted material to disrupt the biofilm integrity and favor thereby the action of antimicrobials. The biomass and the culturable cells of the resulting biofilms at T24 are represented in Fig. 4 and 5. The results of the ANOVA and the multiple comparisons are detailed in Tables S2 to S5. The MICs of the antimicrobials against the reference strains are detailed in Table S6.

Considering first biomass (Fig. 4), the two-way ANOVA analyses indicated that both the enzymes and the antimicrobials caused significant reductions in all biofilm models (Table S2). In many conditions, an additional reduction in biomass was noticed when antimicrobials were applied to biofilms pre-exposed to enzymes (Table S3), although the two-way ANOVA did not generally show a significant interaction (Table S2).

When the biofilms were exposed to enzymes only, the biomass remained reduced at T24 after incubation with subtilisin A in the biofilms, including C. albicans (Fig. 4A–C and G). Biomass was also significantly reduced in three-species and *E.coli:C.albicans* biofilms after incubation with the combination of Ly and the Ce/De/Di cocktail (Fig. 4A and C). When biofilms were exposed to antimicrobials only, the biomass was reduced in all biofilm models except *S.aureus:C.albicans* and S. aureus biofilms (Fig. 4B and E). No significant difference was observed between the two combinations of antimicrobials used (moxifloxacin/caspofungin or meropenem/caspofungin). When assessing the sequential incubation with enzymes and antimicrobials, the reduction in biomass was similar (in *S.aureus:E.coli* biofilm for both types of enzymes; E. coli biofilm for subtilisin A; Ce/De/Di/Ly for C. albicans biofilm [Fig. 4D, F and G]) or even higher (in other conditions) than that observed for enzymes alone. The global efficacy was systematically higher with subtilisin A when the biofilm contained C. albicans, allowing to reduce the biomass of 40% to 90% after exposure to the anti-infective agents.

For the culturable cells (Fig. 5), the two-way ANOVA indicates that the incubation with antimicrobials caused significant reductions in all biofilm models but that the pre-incubation with enzymes only significantly reduced C. albicans culturable cells in dual and single-species biofilms. Globally, the interaction between the two types of incubations was not significant for any biofilm (Table S4).

When the biofilms were incubated with enzymes only, no significant difference with the untreated control was observed at T24 (Fig. 5). On the other hand, when biofilms were incubated with antimicrobials only, the culturable cells were reduced in all biofilm models, with no difference between the combinations of antimicrobials used (Table S5). When comparing the activity of antimicrobials alone to that of the sequential incubation with enzymes and antimicrobials, no gain in activity was noticed, except against C. albicans in dual- and single-species biofilms when the enzyme was subtilisin A (Fig. 5F, I, L).

### Concentration and time effect of the antimicrobials.

To determine whether the potentiation of the antimicrobial activity by the enzymes could be achieved either for lower concentrations of antimicrobials or after shorter incubation times, three-species biofilm pre-exposed or not to enzymes were incubated with antimicrobials at 1 or 10× their MIC (Fig. S3) and for 2 or 6 h (Fig. S4).

When antimicrobials were added at lower concentrations, they were unable to further improve the effects of all enzymes on biomass, as opposed to what was observed when higher concentrations were combined with subtilisin A (compare Fig. S3 A with Fig. 4A). Likewise, the effect of antimicrobials on culturable cells was less important than at higher concentrations and not affected by pre-incubation with enzymes (Fig. S3 B-D). When biofilms were exposed to high concentrations of antimicrobial for shorter times, no further reduction in biomass or culturable cells was observed if biofilm had been preincubated with enzymes (Fig. S4).

### Activity against biofilms formed by clinical isolates.

**FIG 6:**
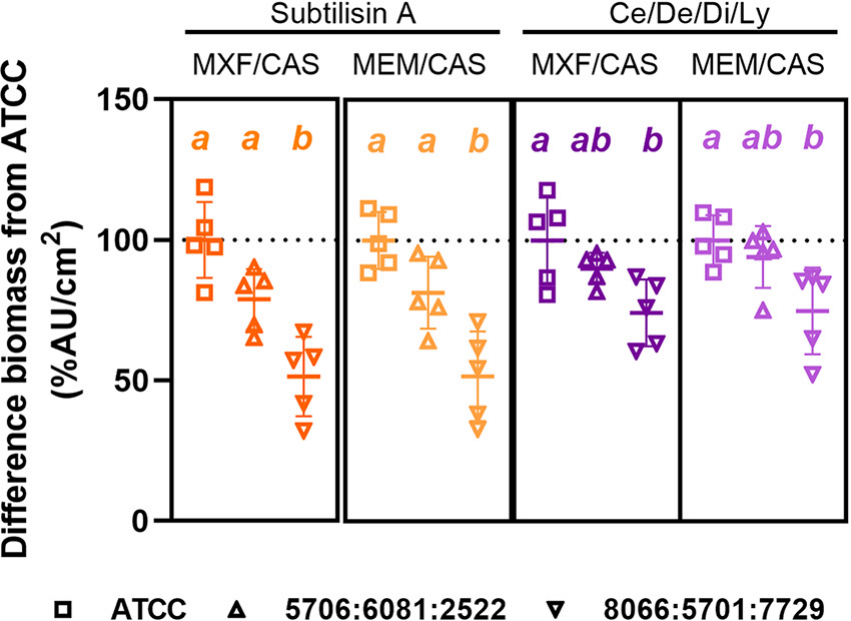
Comparison of the residual biomass of three-species biofilms constructed with reference (ATCC) or clinical isolates (5706:6081:2522 and 8066:5701:7729) grown on Ti coupons after sequential incubation with hydrolytic enzymes during 1 h (subtilisin A 0.5 U/mL [orange] or cellulase 7 U/mL/denarase 250 U/mL/dispersin B 1.25 U/mL/lyticase 12.6 U/mL [Ce/De/Di/Ly, purple]), and antimicrobials during 24 h (moxifloxacin 4 mg/L/caspofungin 13.8 mg/L [MXF/CAS; dark colors] or meropenem 40 mg/L/caspofungin 13.8 mg/L [MEM/CAS; light colors]). Symbols represent the mean of replicates from each of 5 independent experiments. Horizontal bars represent the mean ± SD of all values. Statistical analysis: different lowercase letters with the same color denote significant differences among sets of strains exposed to the same sequential incubation (*P* < 0.05; One-way ANOVA, Tukey posttest).

The model was then extended to clinical isolates and three-species biofilms grown on Ti coupons to examine whether the treatments were also active in a more clinically relevant setting. The biomass of the three-species biofilms formed by references strains or clinical isolates showed no significant difference in control conditions (Fig. S5). The percentage of residual biomass relative to that of biofilms formed by reference strains after a sequential incubation with hydrolytic enzymes and antimicrobials are represented in Fig. 6 and the ANOVA results are detailed in Table S7. The MIC of the antimicrobials used against the clinical strains are detailed in Table S6. In all cases, the reduction in biomass was either similar (isolates 5706:6081:2522) or enhanced by 65% to 40% (isolates 8066:5701:7729) than that observed for the reference strains.

### Biofilm microstructure.

**FIG 7:**
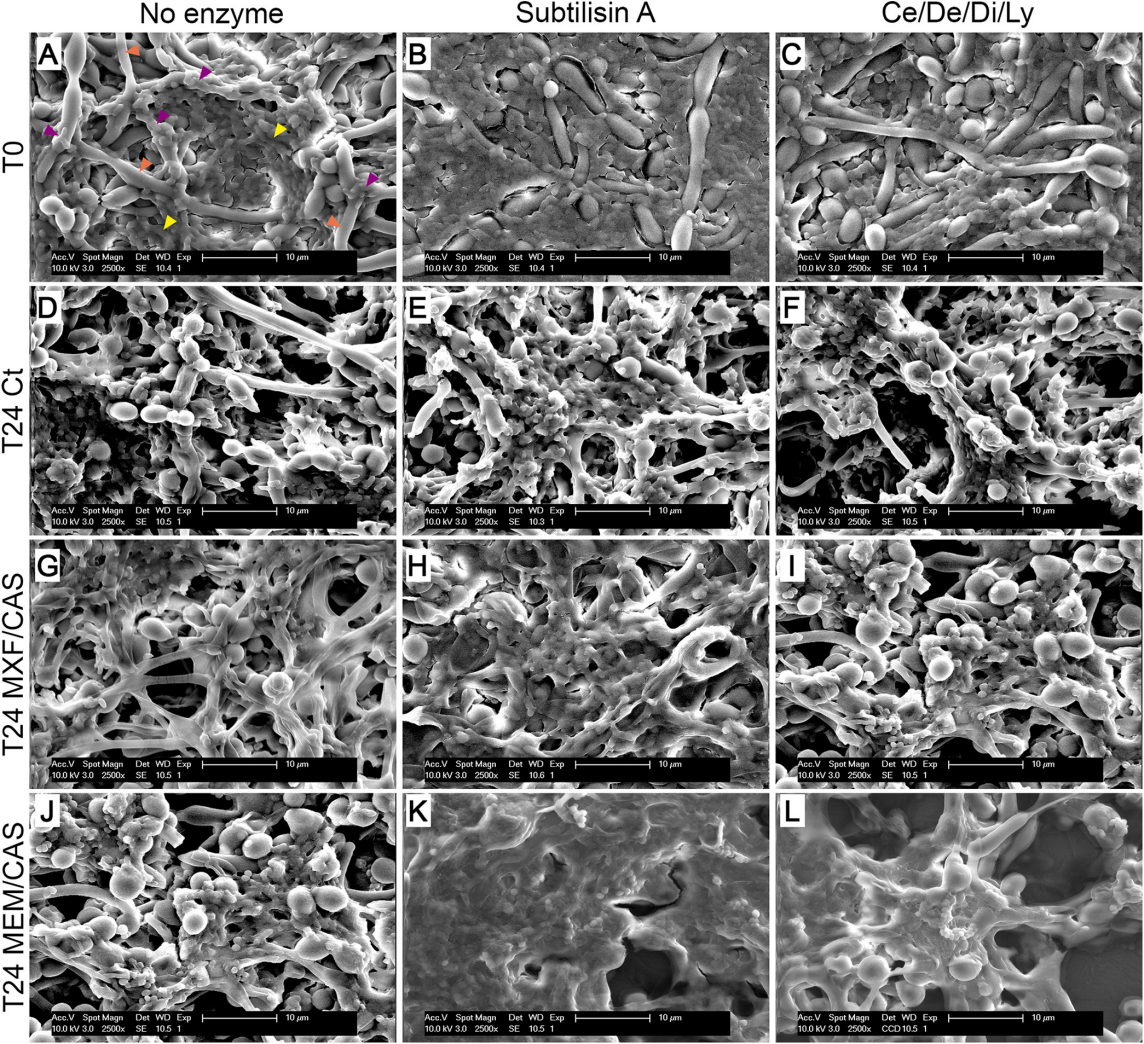
Scanning electron micrographs of three-species biofilms incubated without (left panels) or with subtilisin A 0.5 U/mL (middle panels) or cellulase 7 U/mL/denarase 250 U/mL/dispersin B 1.25 U/mL/lyticase 12.6 U/mL (Ce/De/Di/Ly right panels) for 1 h (T0; A-C) and sequentially incubated in fresh medium (D-F) or with moxifloxacin 4 mg/L/caspofungin 13.8 mg/L (MXF/CAS; G-I) or meropenem 40 mg/L/caspofungin 13.8 mg/L (MEM/CAS; J-L) for 24 h (T24). Orange arrows: C. albicans hyphae. Purple arrows: bacteria on the hyphae. Yellow arrows: bacteria in the matrix. Scale bar 10 μm, magnification ×2500.

**FIG 8:**
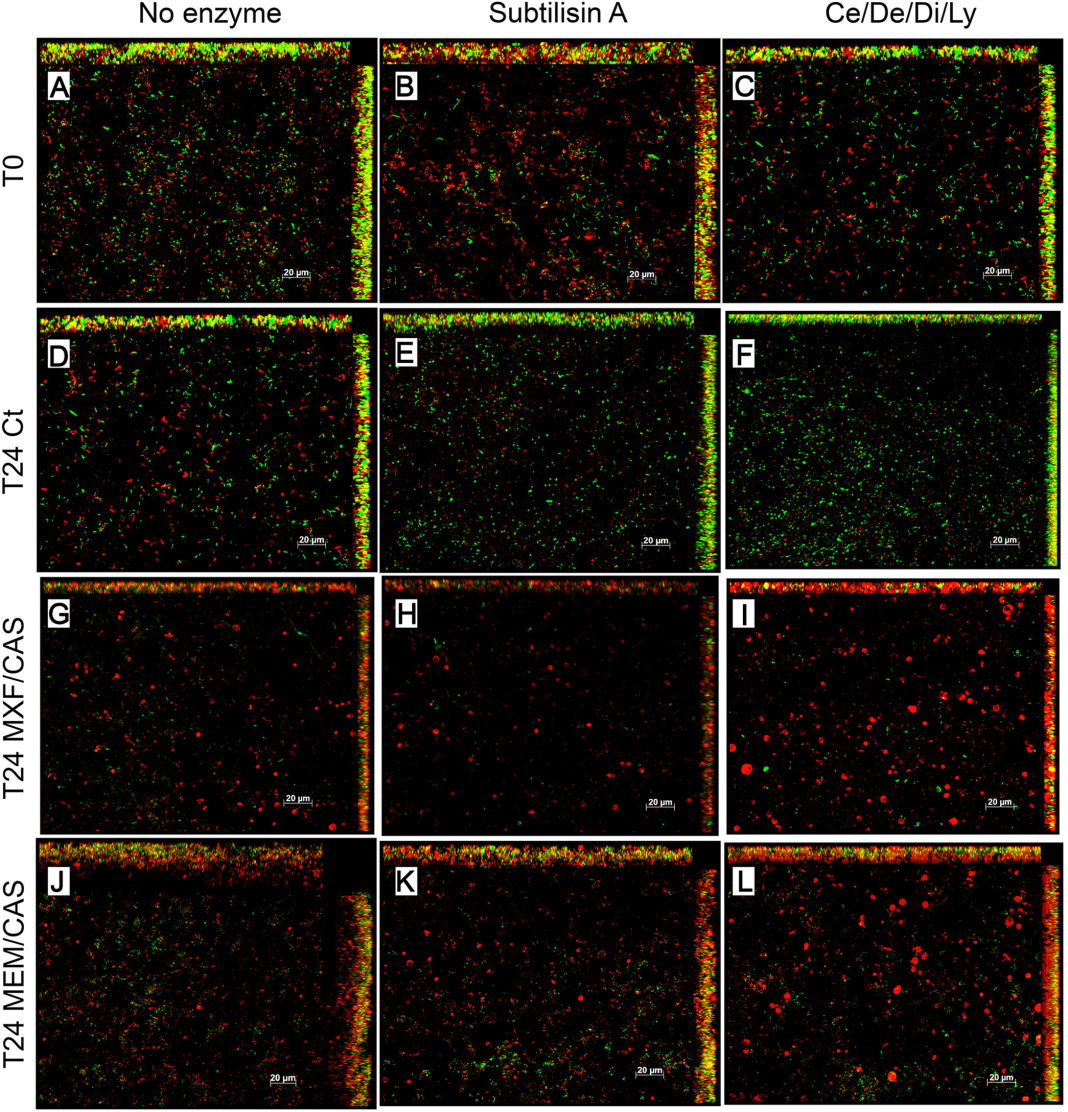
Visualization of three-species biofilms in confocal microscopy (minimal intensity projections) after staining with Syto9 (green; live bacteria) and propidium iodide (red; dead bacteria and fungus). Biofilms were incubated without (left panels) or with subtilisin A 0.5 U/mL (middle panels) or cellulase 7 U/mL/denarase 250 U/mL/dispersin B 1.25 U/mL/lyticase 12.6 U/mL (Ce/De/Di/Ly; right panels) for 1 h (T0; A-C) and sequentially incubated or not with moxifloxacin 4 mg/L/caspofungin 13.8 mg/L (MXF/CAS; G-I) or meropenem 40 mg/L/caspofungin 13.8 mg/L (MEM/CAS; J-L) for 24 h (T24). Scale bar 20 μm.

The microstructure of the biofilms after exposure to enzymes and antimicrobials was visualized by scanning electron microscopy (Fig. 7 and Fig. S6 for images at lower magnification) and confocal microscopy (Fig. 8). At T0, control biofilms (Fig. 7A) appeared as a network of hyphae (orange arrows) with bacterial cells growing on them (purple arrows) and protruding in a densely packed extracellular matrix (yellow arrows). Live/dead pictures show a uniform distribution of bacterial cells (Fig. 8A). Incubation with subtilisin A seemed to scrap the upper layer of the biofilm, unable to liberate the cells deeply embedded in the dense matrix, and to somewhat disorganize the biofilm, leaving a less densely packed biofilm (compare Fig. 7A versus B). This effect was less clear after incubation with the Ce/De/Di/Ly cocktail (compare Fig. 7A versus C). Live/dead pictures show a limited effect of the enzymes on the viability of the bacteria, with a slightly higher abundance of dead cells in red after subtilisin A incubation (compare Fig. 8A versus B).

After 24 h of incubation, the ultrastructure of biofilms looked alike whether preincubated or not with enzymes, with bacteria and matrix closely adhering to the hyphae and similar distribution of live and dead cells (compare panel D versus panels E and F). For control biofilms exposed to antimicrobials for 24 h (Fig. 7G and J), the morphology remained similar. However, the cells appeared coated by the matrix (best seen at lower magnification on Fig. S6) and some cellular debris was visible indicating the death of part of the cells, what is verified with the increased red fluorescence in live/dead staining. Biofilms pre-incubated with enzymes and exposed to moxifloxacin/caspofungin resembled those that were not pre-exposed to the enzymes, except that a diffuse matrix was even more visible around hyphae (compare Fig. 7G versus H and I). Notably, biofilms pre-incubated with enzymes (and especially with subtilisin A) and exposed to meropenem/caspofungin appeared like a deformed amalgam of material covering the cells (compare Fig. 7J versus K and L). Live/dead images showed similar viability for the biofilms pre-incubated with subtilisin A (compare Fig. 8G and J versus H and K). In contrast, the biofilms pre-incubated with Ce/De/Di/Ly showed a higher abundance of large nuclei stained in red, indicating the presence of dead C. albicans cells, which was not present at the same level in biofilms only exposed to the antimicrobials (compare Fig. 8G and J versus I and L) or biofilms only exposed to the enzymes (compare Fig. 8F versus I and L).

## DISCUSSION

This work shows that combining various hydrolytic enzymes capable to degrade matrix constituents with antimicrobials is a useful strategy to act upon interkingdom biofilms. Although this approach has already been described, we extend it here to more complex biofilm models and to enzymes and drug combinations, and we demonstrate the interest in targeting C. albicans specifically when this pathogen is present.

First, in contrast to the pre-established Ce/De/Di cocktail which was unable to reduce the biomass of biofilms comprising C. albicans, subtilisin A and lyticase proved active against fungal biofilms. Subtilisin A is a serine endopeptidase (37). Previously published *in vitro* studies have reported the ability of this enzyme to disrupt the matrix or prevent biofilm formation in S. aureus, E. coli and C. albicans (38–40), but, to the best of our knowledge, this study is the first one to explore its use for the eradication of already established C. albicans biofilms. Lyticase is an endoglycosidase with an affinity toward β(1–3)-glucans (41) found in the matrix and cell wall of fungi but not targeted by the endoglycosidases present in the Ce/De/Di cocktail (cellulase and dispersin B). It has already been shown to reduce C. albicans and P. aeruginosa biofilm biomass *in vitro* (42–44) or to successfully treat a catheter colonized by a *Acremonium* fungus when combined with amphotericin B (45).

Interestingly, subtilisin A alone was as or more effective compared to the Ly/Ce/De/Di cocktail to reduce the biomass for all the biofilm models used here, which would avoid possible proteolytic hydrolysis of other enzymes if he had to be used in combination. On the other hand, the addition of lyticase to the Ce/De/Di cocktail resulted in a limited gain in activity (against E. coli biofilms only), but which still contributes to broadening and maximizing the activity of the cocktail. Yet, the demonstrated cytotoxicity toward osteoblasts of both subtilisin A (previously reported by others [46]) and lyticase may jeopardize their applications *in vivo* if confirmed in more elaborated preclinical toxicity assessments. Of note, the reduction of biomass obtained with subtilisin A as well as with Ly/Ce/De/Di was maintained after 24 h of incubation against three-species biofilms, and for subtilisin A, against dual-species biofilms containing C. albicans, while regrowth was observed for the other models, as previously described for the Ce/De/Di cocktail against single-species bacterial biofilms grown on Titanium alloy coupons (28). This was prevented when the enzymatic incubation was followed by antimicrobials, which allowed to maintain low or even further decrease biofilm biomass. While an effect of antimicrobial agents on culturable cells could be expected, the additional reduction they cause in biomass could potentially be ascribed to the capacity of caspofungin to reduce polysaccharide relative abundance in the matrix (47, 48). Conversely, the antimicrobial activity on culturable cells was generally not potentiated neither impaired by the enzymes, except for a slight improvement against C. albicans culturable cells in single-species and *C.albicans:E:coli* biofilms. Possible reasons could be that biomass is essentially due to C. albicans in biofilm containing this species (34) and that antimicrobial combinations are already quite active on bacteria viability (48).

Electron microscopic images confirm an alteration of the three-species biofilm ultrastructure immediately after enzymatic treatment or after 24 h of incubation with antimicrobials, best seen with subtilisin A and incubation with meropenem/caspofungin. The changes induced resemble those previously described for dental biofilms exposed to a multi-enzyme detergent and a chlorine-containing disinfectant or of *Candida* biofilms exposed to DNases, these changes were interpreted as denoting the reminiscence of microbes in the deepness of the biofilms, or a reduction in EPS, respectively (49, 50).

Confocal microscopy pictures confirm the similar killing trend after incubating with either moxifloxacin/caspofungin or meropenem/caspofungin compared to the one observed in the culturable cells. Interestingly, the pre-incubation with the combination of lyticase with Ce/De/Di markedly increased the abundance of C. albicans cells in the biofilm stained with propidium iodide, thus indicating a disruption of the cell envelope of the fungi. This observation was not evidenced when using the enzymes or the antimicrobials alone. The ability of lyticase to hydrolyze the cell wall glucan is commonly used in the laboratory to form spheroplasts (51). Our hypothesis is that the disruption of the cell wall by lyticase enhances the antimicrobial activity of caspofungin against C. albicans cells. In comparison to the quantification of culturable cells, the pre-incubation with either Ce/De/Di/Ly or subtilisin A also showed a trend toward enhancing C. albicans killing by the antimicrobials. However, it was significant only after pre-incubation with subtilisin A in dual- and single-species biofilms.

Thus, globally, the sequential application of enzymes and antimicrobials remains the most active strategy to reduce both biomass and CFU counts in all types of biofilms. Moreover, the use of antimicrobials is crucial to prevent the dissemination of the pathogens that would have been liberated by the hydrolytic enzymes (20). *In vivo* experiments documented the risk of septicemia in a murine model of wound infection treated by glycoside hydrolyzes to disperse biofilms but also showed that the capacity of antibiotics to prevent septicemia depends on both the biofilm dispersion trigger and of the type of antibiotic used, indicating the importance characterizing the effects of these combinations (26, 52). In our work, we did not quantify planktonic microorganisms potentially released by the enzymatic treatments, but the subsequent incubation with antimicrobials at concentrations that surpass 100× their respective MIC would kill them in our *in vitro* model. *In vivo*, those dispersed microbes could cause an infection of the surrounding soft tissue and enter the bloodstream (26). This type of combinatory therapy with enzymes and antibiotics has however been only rarely exploited in the literature, which essentially reports about the activity of single enzymes combined with single antimicrobials against single-species biofilms (53–55). One study described a synergy between a staphylokinase and a vancomycin-fluconazole combination against *S.aureus:C.albicans* biofilms (56).

In a more clinically oriented perspective, we observed in our complex model, that reducing the time of incubation to 2–6 h or the concentration of antimicrobials to 1-10× MIC abolishes their effects, indicating that the enzymatic treatment does not mitigate the antimicrobial tolerance. Interestingly, however, the activity of a sequential incubation with enzymes and antimicrobials against biofilms formed by clinical isolates on titanium coupons is at least as good as that observed against reference strains, which may encourage further evaluation of these combined treatments in more complex preclinical models. However, the cytotoxicity of subtilisin A and lyticase against osteoblasts is of concern. Alternative enzymes with similar substrate specificity could be considered but are likely to share a similar pattern of toxicity; a more prudent option would consist in limiting the application of these enzymes to infections of tissues where low toxicity could be demonstrated.

Our work still suffers from some limitations, related to the small number of isolates or drug concentrations tested, which is justified by our willingness to rather emphasize a broad selection of enzymes and the direct comparison of mono-, dual- and three-species biofilms. Also, we did not check experimentally the nature of the degradation products resulting from the enzyme application, due to the complex composition of the biofilm matrix. The release of these products could potentially induce a hyper-inflammatory antigenic response and, ultimately, a septic shock (57).

Nevertheless, our results indicate the interest in using enzymes in combination with antimicrobials against hard-to-treat infections as interkingdom biofilms. Even if the activity of antimicrobials was only potentiated against C. albicans, the pre-exposure to enzymes allowed in all cases to reduce biofilm biomass and never led to antagonism. Thus, this work encourages further evaluation of this type of combinatory treatment in clinically relevant infection models.

## MATERIALS AND METHODS

### Strains, growth conditions, and materials.

The reference strains S. aureus ATCC 25923 (MSSA), E. coli ATCC 47076 and C. albicans ATCC 24433 and the clinical isolates S. aureus 5706 (MSSA), S. aureus 8066 (MSSA), E. coli 6081, E. coli 5701, C. albicans 2522 and C. albicans 7729 (all from orthopedic device-associated infections and kindly provided by Prof Rodriguez-Villalobos, *Cliniques Universitaires Saint-Luc*, Brussels, Belgium) were stored in Mueller-Hinton broth + 10% glycerol at −80°C. For all experiments, pre-cultures were prepared from a frozen aliquot on tryptone soy agar (TSA; BD, Franklin Lake, NJ) or Sabouraud glucose agar (SGA; peptone 10 g/L, d-glucose 40 g/L, agar 15 g/L), for the bacteria and C. albicans, respectively, and incubated overnight at 37°C. Aliquots were discarded after thawing.

The biofilms were cultured in two different media based on RPMI 1640 supplemented with l-glutamine (Sigma, Saint Louis, MO), namely, RGP (RPMI + phosphate buffer [KH_2_PO_4_ 50 mM, Na_2_HPO_4_ 74.1 mM, pH 7.4] + 10 g/L of glucose) and RH (RPMI + 25 mM HEPES). They were sterilized by filtration. Inocula were prepared in phosphate buffer saline (PBS; NaCl 137 mM, KCl 2.7 mM, Na_2_HPO_4_ 8 mM, KH_2_PO_4_ 1.5 mM). The selective agar media used for CFU counting were: modified mannitol salt agar (MSA; peptone 5 g/L, NaCl 75 g/L, d-mannitol 10 g/L, agar 15 g/L, amphotericin B 5 mg/L) for S. aureus; selective TSA (TVA; TSA + 5 mg/L vancomycin + 5 mg/L amphotericin B) for E. coli; and selective SGA (S4; SGA + 15 g/L agar, pH 4.5 to prevent bacterial growth) for C. albicans. Antimicrobials were added when the temperature was below 60°C after autoclaving the media.

The hydrolytic enzymes α-amylase from Aspergillus oryzae (EC3.2.1.1), DNase I from bovine pancreas (EC3.1.21.1), lysozyme from chicken egg (EC3.2.1.17), lyticase (Ly) from *Arthrobacter luteus* and Bacillus subtilis and subtilisin A (Su), also called protease, from Bacillus licheniformis (EC3.4.21.62) were obtained from Sigma. Cellulase (Ce) from Aspergillus niger (EC 3.2.1.4, Sigma), denarase from Serratia marcescens (De; c-Lecta GmbH, Leipzig, Germany), and dispersin B from Actinobacillus pleuropneumoniae (Di; Novozymes, Bagsværd, Danemark) were kindly provided by OneLife SA (Louvain-la-Neuve, Belgium).

Moxifloxacin HCl microbiological standard (potency, 97%) was given by Bayer, Leverkusen, Germany. The other drugs were obtained as the corresponding branded products registered for human parenteral use in Belgium or as microbiological standards with the following potencies: Meropenem 100% (Hospira, Brussels, Belgium); vancomycin 100% (Mylan, Hoeilaart, Belgium); caspofungin 50% as Cancidas (MSD, Kenilworth, NJ), cyclosporine 100% and soluble amphotericin B 45% (Sigma-Aldrich). Stock solutions were prepared at 1 mg/mL in sterile ultrapure water for moxifloxacin and meropenem and 2 mg/mL in DMSO for caspofungin and cyclosporine, stored at −20°C and used within 3 weeks. Amphotericin B and vancomycin were prepared in sterile ultrapure water and used immediately.

### MIC.

MICs were determined for moxifloxacin, meropenem and caspofungin using microdilution broth assay. CLSI and EUCAST guidelines were followed for bacteria and C. albicans, respectively, except that we used RGP instead of standard medium (58, 59). For C. albicans, and to avoid a paradoxical growth (48), 1 mg/L of cyclosporine was added to all concentrations of caspofungin. We previously checked that cyclosporine did not interfere in the growth of the microorganisms (48).

### Biofilm culture.

Biofilms were cultured as previously described by Ruiz-Sorribas et al., 2021 (34). Briefly, biofilms were grown in polystyrene Tissue Culture Plates (96 wells-F surface-treated; VWR, Radnor, PA) using as starting inoculum 1.5 × 10^7^ CFU/mL S. aureus in RGP, 6 × 10^6^ CFU/mL E. coli in RGP or 2.5 × 10^6^ CFU/mL C. albicans in RH in order to obtain a stable biofilm at 48 h. The plates were incubated at 37°C without agitation and the medium was renewed daily, which was discarded by aspiration with a pipette. Dual- or three-species biofilms were obtained by precultivating C. albicans biofilms during 24 h in RH before adding S. aureus and/or E. coli at the same inoculums as above in RGP.

For microscopy studies, biofilms were cultivated on Ti alloy disk coupons (Ti-6Al-4V ELI, diameter 12.7 mm, thickness 3.8 mm; BioSurface Technologies, Bozeman, MT) following the same procedure. Coupons were incubated under gentle agitation at 50 rpm. Reconditioning of the coupons was performed using a protocol adapted from (60). In brief, used coupons were immersed in 0.1% (V/V) RBS soap and sonicated for at least 10 min, rinsed in running water and sonicated consecutively in water until no foam was produced, then immersed in 2 M HCl for 2 h, rinsed with ultrapure water, left to dry at 60°C and autoclaved.

### Incubation of biofilms with hydrolytic enzymes.

Preformed biofilms were exposed to hydrolytic enzymes separately or in combination for 1 h in RH at 37°C (T0). α-amylase, cellulase, denarase, DNase I, lysozyme, lyticase and subtilisin A were screened against C. albicans biofilms over a broad range of concentrations. The IC_50_ values were determined as the concentration required to reduce biomass to 50% of the control value. For further experiments, 0.5 U/mL subtilisin A or a mixture of 7 U/mL cellulase, 250 U/mL denarase, 1.25 U/mL dispersin B, and 12.4 U/mL lyticase were used. Note that the concentrations of cellulase, denarase, and dispersin B are lower than those used in our previous study (28) due to regular adaptations of the formula of the cocktail by its industrial provider. All enzymes were preheated for 30 min at 37°C before their use.

### Incubation of biofilms with antimicrobials.

Control biofilms and biofilms pre-exposed to enzymes (T0) were washed once with PBS and exposed to combinations of moxifloxacin/caspofungin or meropenem/caspofungin in RGP at a concentration corresponding to their human *Cmax* (moxifloxacin 4 mg/L, meropenem 40 mg/L, caspofungin 13.8 mg/L) (61–63). Cyclosporine (1 mg/L) was always added to avoid paradoxical growth in the presence of caspofungin (48). Plates were incubated for 24 h at 37°C (T24).

### Culturable cells from biofilms.

As previously described, the medium was removed by pipetting at the end of the incubation period, the biofilms were washed once with PBS and detached by mechanically scratching the surface with an inoculation loop, resuspended in 200 μL of PBS with vigorous pipetting, and disaggregated by sonication (Q700, QSonica, Newton, Connecticut) at 60% amplitude for 30 s directly in the well (34). The content of two wells was pooled and diluted appropriately. Fifty μL of suspension or dilutions thereof were spread on agar plates (using selective media for multi-species biofilms). Colonies on TSA or TVA were counted after overnight incubation. Colonies on SGA or S4 plates were counted after 24 h, those on MSA, after 48 h. The CFU were normalized per cm^2^ of surface (available surface: 1.57 cm^2^ for each microplate well [diameter 0.64 cm and culture volume 200 μL]) and expressed as the difference from the control at time zero (T0) as (CFU_sample_/surface) – (CFU_control [T0]_/surface).

### Biomass assay.

Total biomass was estimated using a previously described protocol (34). Briefly, after removal of the medium, biofilms were dried at 60°C and stained by 200 μL of crystal violet (Sigma) at 0.5% (V/V, final concentration 115 mg/L) in water for 10 min at room temperature. The dye in excess was eliminated by rinsing with running water. Bound crystal violet was resolubilized in 200 μL of acetic acid 66% (V/V) (Merck, Darmstadt, Germany) in water for at least 1 h in darkness and quantified by measure of the absorbance at 570 nm using a microplate reader (SpectraMax Gemini XS microplate spectrophotometer; Molecular Devices LLC, San José, CA). The absorbance was normalized per cm^2^ of surface (available surface: 1.57 cm^2^ for each microplate well [diameter 0.64 cm and culture volume 200 μL] or 1.27 cm^2^ for each coupon [diameter 1.27 cm]) and expressed as the percentage of the control at Time zero (T0) as [(Abs_sample_/surface)_/_(Abs_control [T0]_/surface)] * 100.

### Scanning electron microscopy.

Scanning electron micrographs of biofilms were taken following a protocol adapted from Ruiz-Sorribas et al., 2021 (34). Briefly, biofilms were fixed with 2.5% glutaraldehyde (Sigma) in sodium cacodylate buffer 0.1 M at pH 7.4 (Sigma) for 30 min, washed in PBS and dehydrated by successive incubations of 20 min each in 30, 50, 70, 90% ethanol, then three times 100% ethanol (Merck). After drying at room temperature, biofilms were coated with Pt/Pd 80/20 using a sputtering device (Quorum Q150T S, Quorum Technologies, Laughton, UK) and visualized using a scanning electron microscope (FEI XL30-FEG, FEI, Hillsboro, OR) at high-vacuum with a 10 keV acceleration voltage.

### Confocal microscopy.

Biofilms were imaged after a live/dead staining following a protocol detailed in Ruiz-Sorribas et al. (48). Briefly, 200 μL of FilmTracer Live/Dead Staining (Syto9 0.01 mM, propidium iodide 0.06 mM; Invitrogen, Carlsbad, CA) in water was added very gently on top of the biofilms and incubated 30 min in darkness. Excess of dyes was rinsed with water and excess liquid was removed with absorbent paper. Coupons were mounted with Dako mounting oil (Agilent, Santa Clara, CA) and a glass coverslip. Z-stacks pictures of the stained biofilms were taken with an AxioImager.z1-ApoTome microscope (Carl Zeiss, Oberkochen, Germany) through a multi-acquisition from the top to the bottom of the biofilm. The means of filter sets (excitation/emission) were green 460/550 nm and red 535/590 nm. Pictures were analyzed and converted to maximal intensity projections with AxioVision Rel. 4.8.2.0 (Carl Zeiss).

### Haemolysis and cytotoxicity.

The hemolytic activity of cellulase, denarase, dispersin B, lyticase and subtilisin A was tested by quantifying the release of hemoglobin from sheep red blood cells (Sigma) using a protocol adapted from Ruiz et al. 2017 (64). The blood + 1 mg/mL EDTA was centrifuged (10 min, 500 g, 4°C). The supernatant was discarded, and the pellet was washed twice with HEPES buffer (HEPES 5 mM, NaCl 150 mM, pH 7.4), and diluted 1/10 in the same buffer. 100 μL of red blood cells + 100 μL of preheated enzymes in RH were incubated in Cellstar U-bottom 96-well suspension culture plates (Greiner Bio-One, Kremsmunster, Austria) for 1 h at 37°C. After incubation, the plates were centrifuged (10 min, 500 g, 4°C); 100 μL of supernatant was diluted with 100 μL HEPES buffer to measure the absorbance at 540 nm. Haemolysis was calculated as the percentage of the absorbance of the sample versus that of full hemolysis control (solution of Triton-X 1%): (Abs_sample_/Abs_control [Triton-X]_) * 100.

The cytotoxicity of cellulase, denarase, dispersin B, lyticase, and subtilisin A against the adherent cell lines MG63 osteoblasts and J774 macrophages and against THP-1 monocytes growing in suspension was tested using the MTT assay, based on a protocol adapted from Bergidge et al. 2005 (65). All cell lines were inoculated at 10^5^ cells/well in 96-wells microtiter plates and incubated for 24 h at 37°C in the appropriate conditions (osteoblasts, in DMEM + 10% FBS and macrophages, in RPMI + 10% FBS in F-bottom plates; monocytes, in RPMI + 10% FBS using U-bottom plates). After incubation, adherent cell lines were exposed to 200 μL of preheated enzymes in RH for 1 h at 37°C and washed twice with PBS, then incubated with 100 μL MTT 500 μg/mL for 1 h at 37°C in darkness. The MTT solution was discarded and the formazan crystals were dissolved in 200 μL DMSO for 10 min at 37°C in darkness. The DMSO solution was then transferred to a new F-bottom plate and the absorbance at 540 nm was quantified. For monocytes, an additional step of centrifugation (500 g, 5 min) was added before any removal of the media from the plate. Cytotoxicity was calculated as [1 - (Abs_sample_/Abs_control [no enzyme]_)] * 100.

### Data and statistical analysis.

GraphPad Prism 8.0.2 (GraphPad Software, San Diego, CA) was used to plot the results and perform the statistical analyses.

### Data availability.

All data will be made available from the corresponding author upon request.
