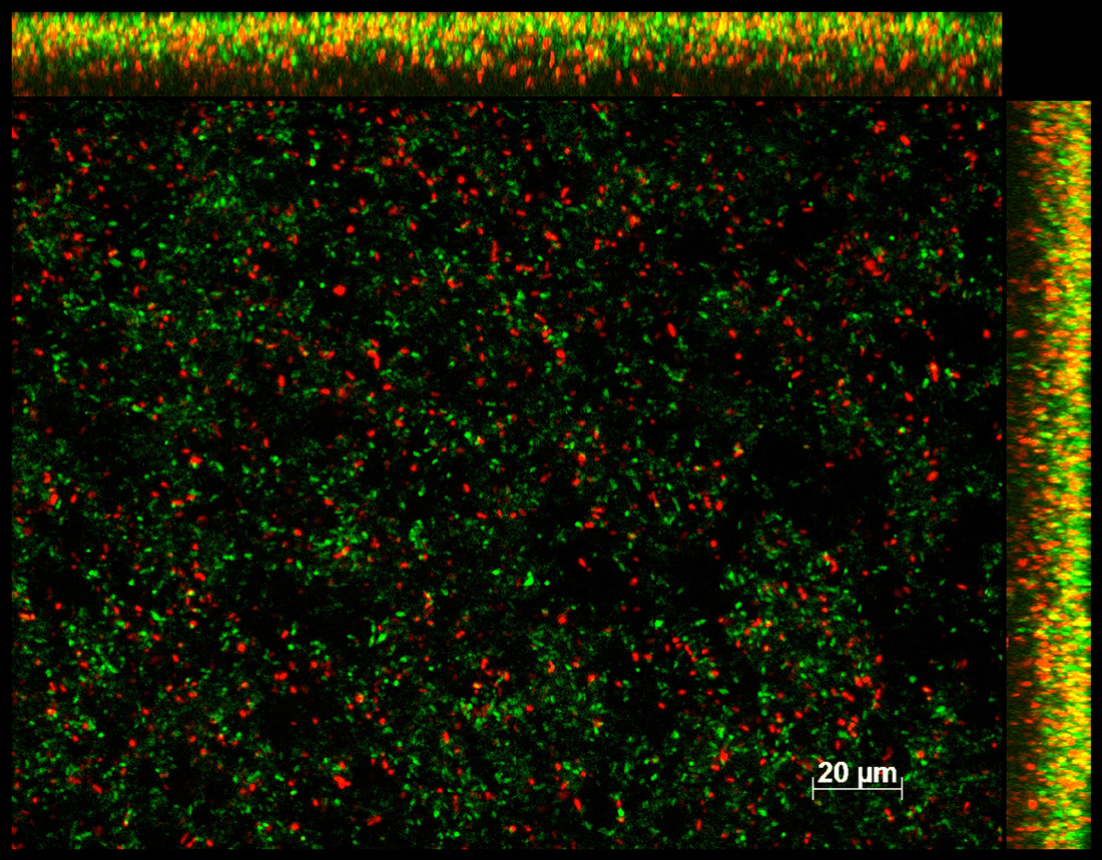# Correction for Ruiz-Sorribas et al., “Hydrolytic Enzymes as Potentiators of Antimicrobials against an Inter-Kingdom Biofilm Model”

**DOI:** 10.1128/spectrum.04671-22

**Published:** 2022-12-14

**Authors:** Albert Ruiz-Sorribas, Hervé Poilvache, Nur Hidayatul Nazirah Kamarudin, Annabel Braem, Françoise Van Bambeke

**Affiliations:** American Society for Microbiology

## AUTHOR CORRECTION

Volume 10, no. 1, e02589-21, 2022, https://doi.org/10.1128/spectrum.02589-21. Page 9, Fig. 7: Panel J is an inadvertent duplicate of panel I; it should appear as shown below.

Page 10, Fig. 8: Panel D is an inadvertent duplicate of panel C; it should appear as shown below.

**FIG 7:**
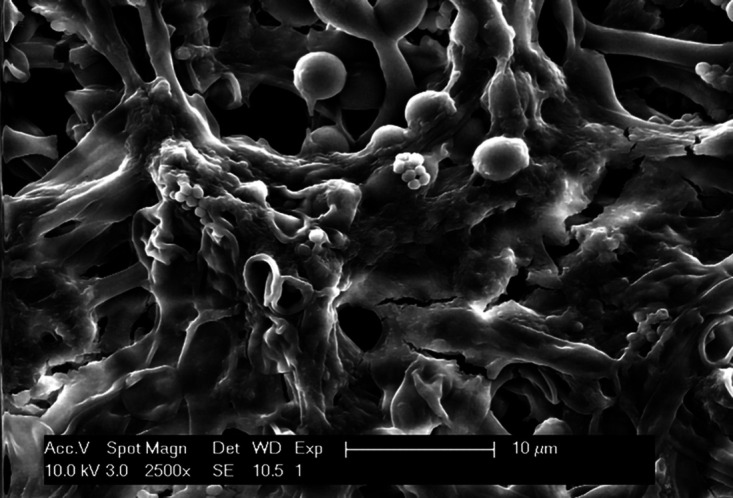


**FIG 8:**