# Supplementary material for: Hydrolytic Enzymes as Potentiators of Antimicrobials against an Inter-Kingdom Biofilm Model
Source: Microbiol Spectr. 2022 Feb 23;10(1):e02589-21. doi: 10.1128/spectrum.02589-21 (PMC8865531; doi:10.1128/spectrum.02589-21)
Supplement: SUPPLEMENTAL FILE 1 — Supplemental material. Download SPECTRUM02589-21_Supp_1_seq4.pdf, PDF file, 2.1 MB [file spectrum02589-21_supp_1_seq4.pdf]

Supplementary information

Hydrolytic enzymes as potentiators of antimicrobials against an inter-kingdom biofilm model

Albert Ruiz-Sorribas [a], Hervé Poilvache [bc], Nur Hidayatul Nazirah Kamarudin[d,e], Annabel Braem[d] and Françoise Van Bambeke [a]

[a] Pharmacologie cellulaire et moléculaire, Louvain Drug Research Institute, Université catholique de Louvain, Brussels, Belgium.

[b] Laboratoire de neuro musculo squelettique, Institut de Recherche Expérimentale et Clinique, Université catholique de Louvain, Brussels, Belgium.

[c] Orthopaedic surgery department, Cliniques Universitaires Saint-Luc, Brussels, Belgium.

[d] Department of Materials Engineering, Biomaterials and Tissue Engineering research group, KU Leuven, Leuven, Belgium.

[e] Department of Chemical and Process Engineering, Faculty of Engineering and Built Environment, Universiti Kebangsaan Malaysia, Bangi, Malaysia.

Corresponding author: Françoise Van Bambeke (francoise.vanbambeke@uclouvain.be)

Figure S1. Time-effect of 12.6 U/mL lyticase (orange) or 0.5U/mL subtilisin A (blue) against *C. albicans* biofilm biomass. Data are the mean ± SD of triplicates and expressed in percentage of the control values (untreated biofilms).

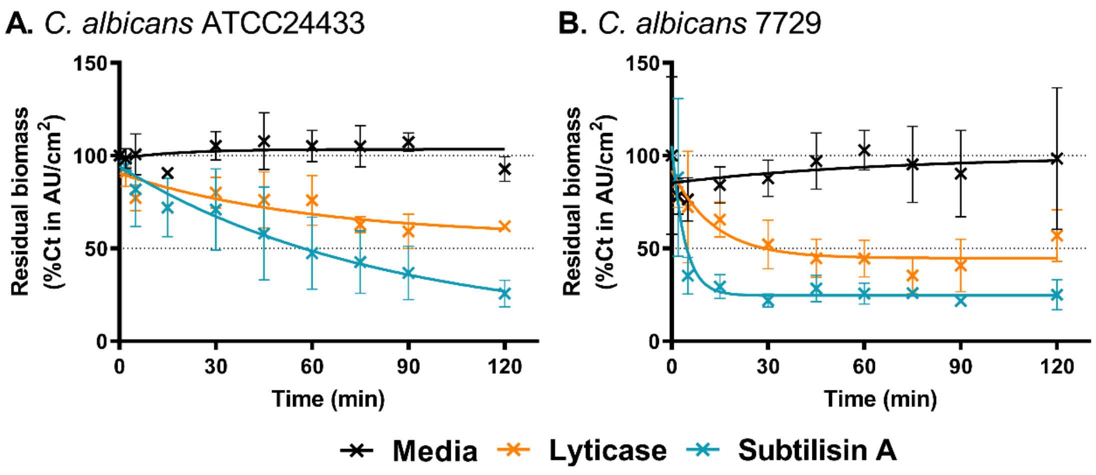

Figure S2. Culturable cells reduction of biofilms from different species after 1 h incubation with hydrolytic enzymes (corresponding to T0 in further experiments). The enzymes tested are subtilisin A 0.5 U/mL (orange) or cellulase 7 U/mL / denarase 250 U/mL / dispersin B 1.25 U/mL / lyticase 12.6 U/mL (Ce/De/Di/Ly, purple) versus no enzyme (black). Symbols represent the mean of replicates from each of 4 independent experiments. Horizontal bars represent the mean ± SD of all values. The horizontal dotted line represents the initial value (T0). Statistical analysis: Conditions with different letters are significantly different from one another ($p<0.05$; one-way ANOVA, Tukey post-test).

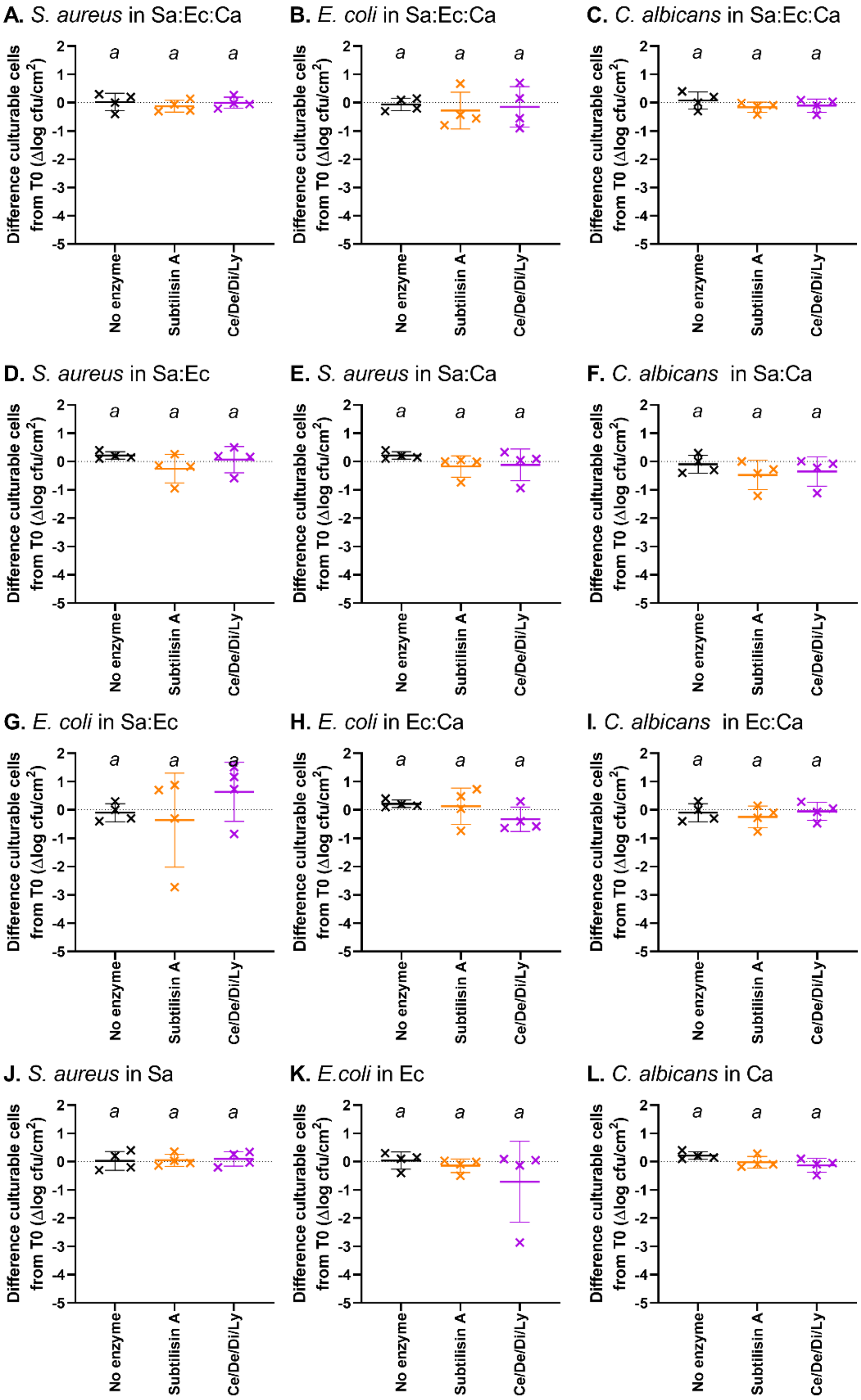
A. S. aureus in Sa:Ec:Ca
B. E. coli in Sa:Ec:Ca
C. C. albicans in Sa:Ec:Ca
D. S. aureus in Sa:Ec
E. S. aureus in Sa:Ca
F. C. albicans in Sa:Ca
G. E. coli in Sa:Ec
H. E. coli in Ec:Ca
I. C. albicans in Ec:Ca
J. S. aureus in Sa
K. E.coli in Ec
L. C. albicans in Ca
Difference culturable cells from T0 (Δlog cfu/cm2)
No enzyme
Subtilisin A
Ce/De/Di/Ly
a

Figure S3. Concentration-effect of sequential incubation in control conditions (black) or with hydrolytic enzymes, subtilisin A 0.5 U/mL (orange) or cellulase 7 U/mL / denarase 250 U/mL / dispersin B 1.25 U/mL / lyticase 12.6 U/mL (Ce/De/Di/Ly, purple) during 1 h followed by 24 incubation in the absence or in the presence of antimicrobials, moxifloxacin / caspofungin (MXF/CAS) or meropenem / caspofungin (MEM/CAS), against three-species biofilm biomass (A) and culturable cells of *S. aureus* (B), *E. coli* (C) and *C. albicans* (D). $MIC_{MXF\ vs.\ bacteria}$= 0.06 mg/L. $MIC_{MEM\ vs.\ bacteria}$= 0.03 mg/L. $MIC_{CAS\ vs.\ C.\ albicans}$= 0.125 mg/L. Symbols represent the mean of replicates from each of 3 independent experiments. Horizontal bars represent the mean ± SD of all values. The horizontal dotted line represents the initial value (T0). Data are expressed as percentage (A) or as difference (B-D) of the value measured at the end of the preincubation with enzymes (T0). Statistical analysis: different lowercase letters of same greyscale colour denote significant differences among enzymes for each antimicrobial incubation; different uppercase letters of the same colour denote significant differences among antimicrobials for each enzymatic pre-incubation ($p<0.05$; Two-way ANOVA, Tukey post-test).

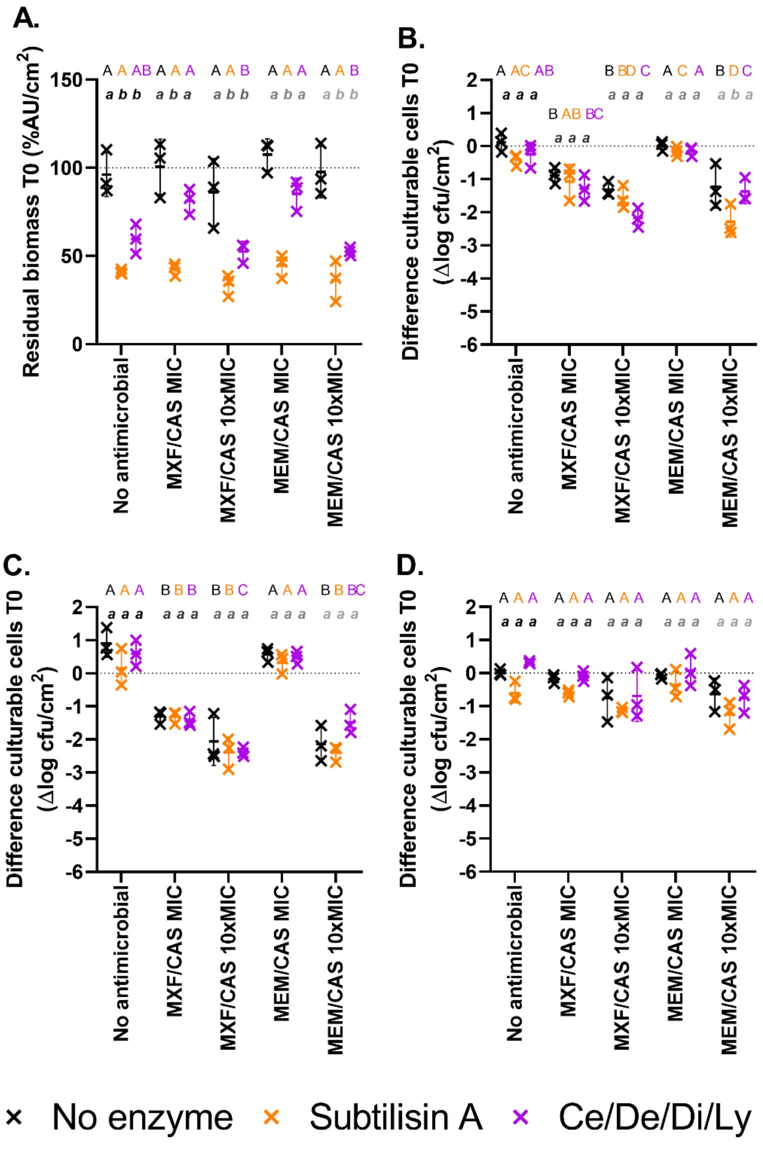

Figure S4. Effect of sequential incubation with hydrolytic enzymes, subtilisin A 0.5 U/mL (orange) or cellulase 7 U/mL / denarase 250 U/mL / dispersin B 1.25 U/mL / lyticase 12.6 U/mL (Ce/De/Di/Ly, purple) or no enzyme (black) during 1 h, and antimicrobials, moxifloxacin 4 mg/L / caspofungin 13.8 mg/L (MXF/CAS) or meropenem 40 mg/L / caspofungin 13.8 mg/L (MEM/CAS) during 2 or 6 h, on three-species biofilm biomass (A) and culturable cells of *S. aureus* (B), *E. coli* (C) and *C. albicans* (D). Symbols represent the mean of replicates from each of 3 independent experiments. Horizontal bars represent the mean ± SD of all values. The horizontal dotted line represents the initial value (T0). Data are expressed as percentage (A) or as difference (B-D) of the value measured at the end of the preincubation with enzymes (T0). Statistical analysis: different lowercase letters of same greyscale colour denote significant differences among enzymes for each antimicrobial treatment; different uppercase letters of same colour denote significant differences among antimicrobials for each enzymatic pre-treatment ($p<0.05$; two-way ANOVA, Tukey post-test).

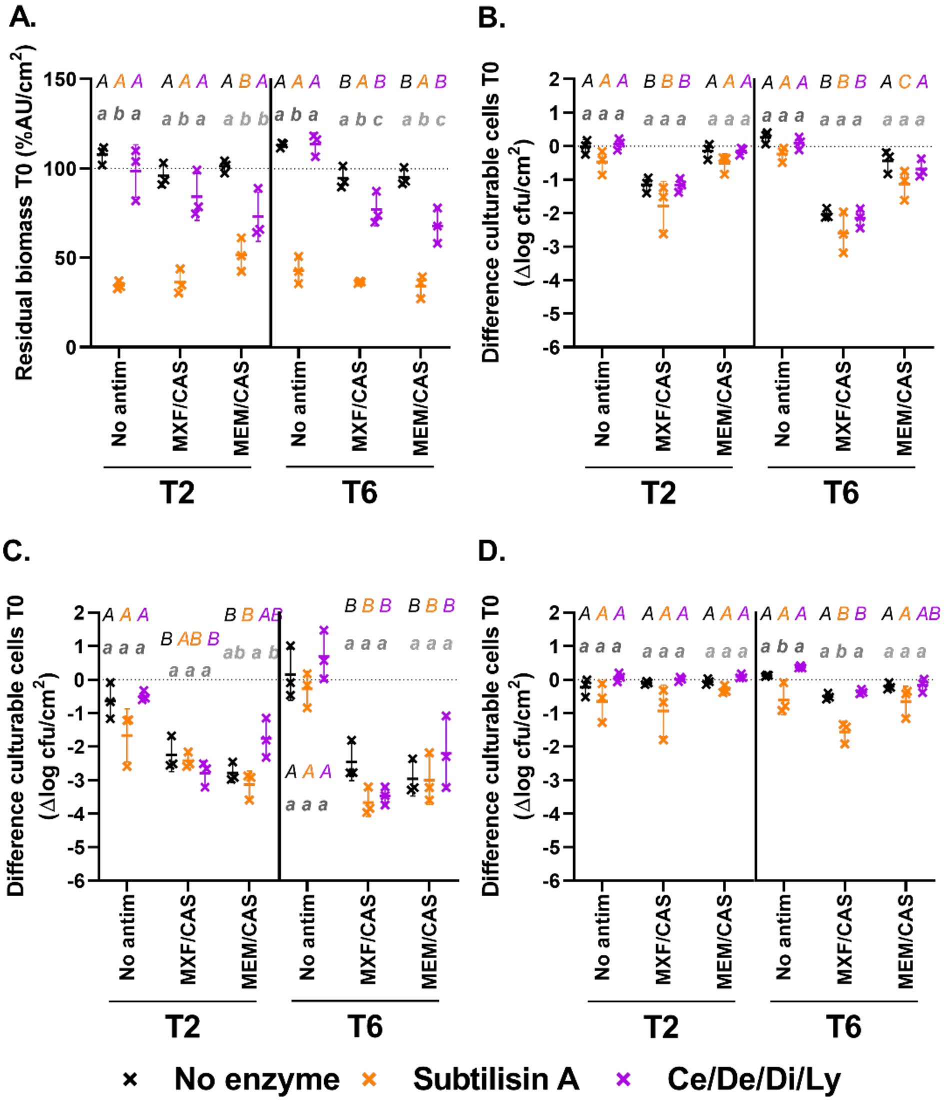

Figure S5. Biomass of biofilms grown in Ti coupons using reference and clinical strains without enzymes nor antimicrobials. Symbols represent the mean of replicates from each of 3 independent experiments. Horizontal bars represent the mean ± SD of all values. Conditions with different letters are significantly different from one another ($p<0.05$; non-parametric one-way ANOVA, Tukey post-test).

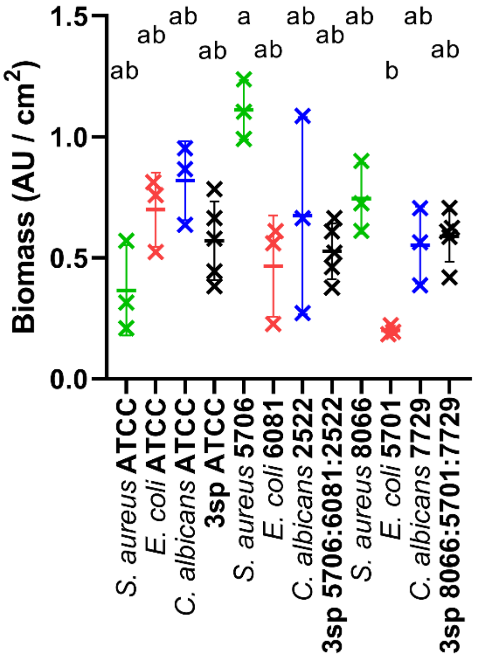

Figure S6. Scanning electron micrographs of three-species biofilms incubated with or without subtilisin A 0.5 U/mL or cellulase 7 U/mL / denarase 250 U/mL / dispersin B 1.25 U/mL / lyticase 12.6 U/mL (Ce/De/Di/Ly) for 1 h (T0) and sequentially incubated or not with moxifloxacin 4 mg/L / caspofungin 13.8 mg/L (MXF/CAS) or meropenem 40 mg/L / caspofungin 13.8 mg/L (MEM/CAS) for 24 h (T24). Scale bar 50 µm, magnification 650x.

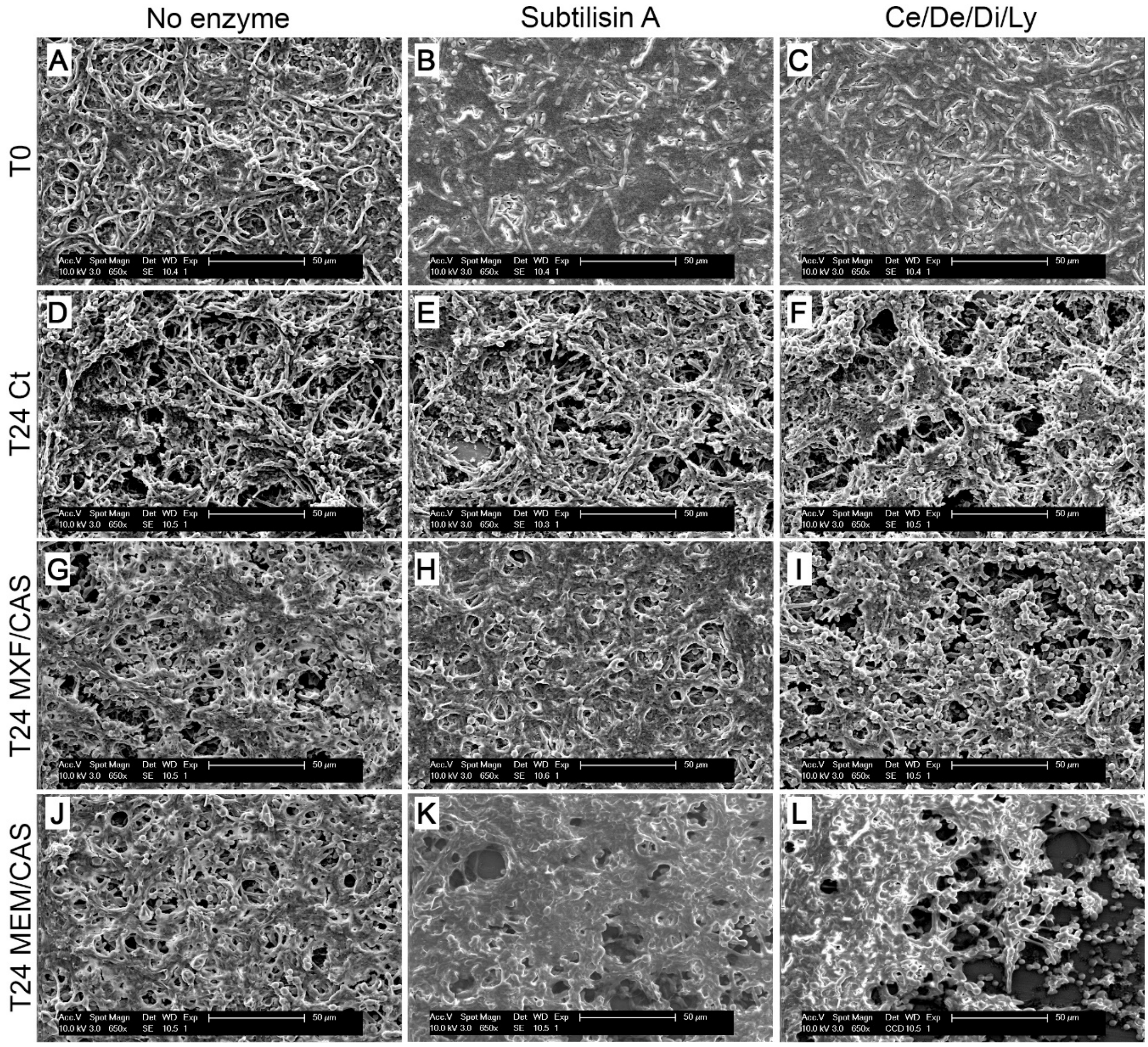

Table S1. One-way ANOVA results from figure 3. p-value of Tukey's multiple comparisons test. p-values lower than 0.05 are represented in red.

	S.aureus: E.coli: C.albicans	*S.aureus: C.albicans*	*E.coli: C.albicans*	*S.aureus: E.coli*	*S. aureus*	*E. coli*	*C. albicans*
No enzyme vs. Lyticase	0.007	<0.001	0.007	0.005	0.003	0.55	0.32
No enzyme vs. Subtilisin A	<0.001	<0.001	<0.001	<0.001	0.01	0.002	<0.001
No enzyme vs. Ce/De/Di	>0.99	>0.99	>0.99	0.45	0.11	0.008	>0.99
No enzyme vs. Ce/De/Di/Ly	0.97	0.17	0.06	<0.001	0.05	<0.001	0.02
No enzyme vs. Ce/De/Di/Su	<0.001	<0.001	<0.001	<0.001	0.009	<0.001	<0.001
No enzyme vs. Ce/De/Di/Ly/Su	<0.001	<0.001	<0.001	0.002	0.006	<0.001	<0.001
Lyticase vs. Subtilisin A	<0.001	<0.001	<0.001	>0.99	>0.99	0.09	0.004
Lyticase vs. Ce/De/Di	0.11	0.001	0.02	0.56	0.98	0.19	0.19
Lyticase vs. Ce/De/Di/Ly	0.07	0.30	0.96	0.98	0.95	<0.001	0.90
Lyticase vs. Ce/De/Di/Su	0.001	0.007	<0.001	0.63	>0.99	<0.001	0.08
Lyticase vs. Ce/De/Di/Ly/Su	0.003	0.007	<0.001	0.93	>0.99	<0.001	<0.001
Subtilisin A vs. Ce/De/Di	<0.001	<0.001	<0.001	0.23	>0.99	>0.99	<0.001
Subtilisin A vs. Ce/De/Di/Ly	<0.001	<0.001	<0.001	>0.99	>0.99	0.23	0.11
Subtilisin A vs. Ce/De/Di/Su	0.70	0.90	0.98	0.90	0.98	0.26	0.99
Subtilisin A vs. Ce/De/Di/Ly/Su	0.52	0.90	>0.99	>0.99	0.96	0.12	0.97
Ce/De/Di vs. Ce/De/Di/Ly	>0.99	0.11	0.12	0.18	>0.99	0.26	0.01
Ce/De/Di vs. Ce/De/Di/Su	<0.001	<0.001	<0.001	0.06	0.95	0.28	<0.001
Ce/De/Di vs. Ce/De/Di/Ly/Su	<0.001	<0.001	<0.001	0.18	0.92	0.13	<0.001
Ce/De/Di/Ly vs. Ce/De/Di/Su	<0.001	<0.001	<0.001	0.95	0.90	>0.99	0.58
Ce/De/Di/Ly vs. Ce/De/Di/Ly/Su	<0.001	<0.001	<0.001	>0.99	0.85	0.98	0.02
Ce/De/Di/Su vs. Ce/De/Di/Ly/Su	>0.99	>0.99	0.92	>0.99	>0.99	>0.99	0.75

Table S2. Two-way ANOVA results from figure 4. p-values lower than 0.05 are represented in red.

	S.aureus:E.coli: C.albicans		*S.aureus:C.albicans*		*E.coli:C.albicans*		*S.aureus:E.coli*		*S. aureus*		*E. coli*		*C. albicans*	
Source of Variation	% of total variation	P value	% of total variation	P value	% of total variation	P value	% of total variation	P value	% of total variation	P value	% of total variation	P value	% of total variation	P value
Interaction	1.85	0.09	5.29	0.06	0.442	0.86	2.71	0.54	13.5	0.17	5.65	0.31	2.19	0.33
Antimicrobials	12.8	<0.001	11.1	<0.001	22.9	<0.001	72.6	<0.001	19.2	0.02	60.8	<0.001	47.6	<0.001
Enzyme	75.7	<0.001	77.8	<0.001	70.5	<0.001	9.54	0.01	33.2	0.002	13.7	0.009	42.2	<0.001

Table S3. p-value of Tukey's multiple comparison of two-way ANOVA of figure 4. p-values lower than 0.05 are represented in red.

Within each enzyme, compare antimicrobials		*S.aureus: E.coli: C.albicans*	*S.aureus: C.albicans*	*E.coli: C.albicans*	*S.aureus: E.coli*	*S. aureus*	*E. coli*	*C. albicans*
No enzyme	No antim vs. MXF/CAS	<0.001	0.16	<0.001	<0.001	>0.99	0.001	<0.001
	No antim vs. MEM/CAS	<0.001	0.27	<0.001	<0.001	0.91	<0.001	0.003
	MXF/CAS vs. MEM/CAS	0.97	0.94	0.94	0.64	0.94	0.96	0.67
Subtilisin A	No antim vs. MXF/CAS	<0.001	0.32	0.009	<0.001	0.06	0.004	<0.001
	No antim vs. MEM/CAS	0.002	0.67	0.01	0.009	0.1	0.04	<0.001
	MXF/CAS vs. MEM/CAS	0.79	0.81	>0.99	0.3	0.97	0.49	0.91
Ce/De/Di/Ly	No antim vs. MXF/CAS	0.09	<0.001	0.001	<0.001	0.05	0.001	<0.001
	No antim vs. MEM/CAS	0.12	<0.001	0.003	0.07	0.03	0.16	0.002
	MXF/CAS vs. MEM/CAS	>0.99	0.92	0.91	0.04	0.94	0.06	0.93

Within each antimicrobial, compare enzymes		*S.aureus: E.coli: C.albicans*	*S.aureus: C.albicans*	*E.coli: C.albicans*	*S.aureus: E.coli*	*S. aureus*	*E. coli*	*C. albicans*
No antim	No enzyme vs. Subtilisin A	<0.001	<0.001	<0.001	0.1	0.78	0.42	0.004
	No enzyme vs. Ce/De/Di/Ly	<0.001	>0.99	<0.001	0.03	0.9	0.03	0.68
	Subtilisin A vs. Ce/De/Di/Ly	0.006	<0.001	<0.001	0.81	0.52	0.3	0.03
MXF/CAS	No enzyme vs. Subtilisin A	<0.001	<0.001	<0.001	0.34	0.01	0.74	<0.001
	No enzyme vs. Ce/De/Di/Ly	<0.001	0.003	0.002	0.1	0.1	0.03	0.83
	Subtilisin A vs. Ce/De/Di/Ly	<0.001	<0.001	0.006	0.73	0.6	0.11	<0.001
MEM/CAS	No enzyme vs. Subtilisin A	<0.001	<0.001	<0.001	0.7	0.01	0.77	<0.001
	No enzyme vs. Ce/De/Di/Ly	<0.001	0.003	0.002	0.9	0.03	0.99	0.53
	Subtilisin A vs. Ce/De/Di/Ly	<0.001	<0.001	0.003	0.93	0.91	0.68	<0.001

Table S4. Two-way ANOVA results from figure 5. p-values lower than 0.05 are represented in red.

	S. aureus in Sa:Ec:Ca		*E. coli* in Sa:Ec:Ca		*C.albicans* in Sa:Ec:Ca		*S. aureus* in Sa:Ca		*C.albicans* in Sa:Ca		*E. coli* in Ec:Ca		*C.albicans* inEc:Ca	
Source of Variation	% of total variation	P value	% of total variation	P value	% of total variation	P value	% of total variation	P value	% of total variation	P value	% of total variation	P value	% of total variation	P value
Interaction	0.475	0.97	0.199	0.97	2.54	0.45	1.76	0.56	3.31	0.35	0.794	0.57	5.83	0.27
Antimicrobial	74.5	<0.001	82.9	<0.001	65.4	<0.001	86.7	<0.001	74.4	<0.001	93	<0.001	55.8	<0.001
Enzyme	1.95	0.33	0.373	0.63	4.42	0.06	1.24	0.36	9.78	0.005	1.41	0.1	19.9	0.001

	S. aureus in Sa:Ec		*E. coli* in Sa:Ec		*S. aureus* in Sa		*E. coli* in Ec		*C.albicans* in Ca	
Source of Variation	% of total variation	P value	% of total variation	P value	% of total variation	P value	% of total variation	P value	% of total variation	P value
Interaction	0.711	0.9	1.23	0.82	2.75	0.63	1.05	0.25	2.98	0.39
Antimicrobial	86.9	<0.001	84.2	<0.001	77.2	<0.001	95.2	<0.001	73.5	<0.001
Enzyme	0.0652	0.95	0.147	0.91	1.31	0.55	0.6	0.21	11.2	0.003

Table S5. p-value of Tukey's multiple comparison of two-way ANOVA of figure 5. p-values lower than 0.05 are represented in red.

Within each enzyme, compare antimicrobials		*S. aureus* in Sa:Ec:Ca	*E. coli* in Sa:Ec:Ca	*C.albicans* in Sa:Ec:Ca	*S. aureus* in Sa:Ca	*C.albicans* in Sa:Ca	*E. coli* in Ec:Ca	*C.albicans* inEc:Ca	*S. aureus* in Sa:Ec	*E. coli* in Sa:Ec	*S. aureus* in Sa	*E. coli* in Ec	*C.albicans* in Ca
No enzyme	No antim vs. MXF/CAS	<0.001	<0.001	<0.001	<0.001	0.001	<0.001	0.06	<0.001	0.002	0.003	<0.001	<0.001
	No antim vs. MEM/CAS	0.003	<0.001	<0.001	<0.001	0.002	<0.001	0.13	<0.001	<0.001	<0.001	<0.001	0.006
	MXF/CAS vs. MEM/CAS	0.53	>0.99	0.85	0.41	0.98	0.69	0.91	0.9	0.35	0.7	>0.99	0.68
Subtilisin A	No antim vs. MXF/CAS	<0.001	<0.001	<0.001	<0.001	<0.001	<0.001	0.004	<0.001	<0.001	<0.001	<0.001	<0.001
	No antim vs. MEM/CAS	0.003	<0.001	0.01	<0.001	0.003	<0.001	0.007	<0.001	<0.001	0.003	<0.001	<0.001
	MXF/CAS vs. MEM/CAS	0.2	0.8	0.02	0.35	0.1	0.4	0.98	0.72	0.98	0.77	0.98	0.43
Ce/De/Di/Ly	No antim vs. MXF/CAS	<0.001	<0.001	<0.001	<0.001	<0.001	<0.001	<0.001	<0.001	<0.001	<0.001	<0.001	<0.001
	No antim vs. MEM/CAS	<0.001	<0.001	<0.001	<0.001	<0.001	<0.001	<0.001	<0.001	<0.001	0.005	<0.001	<0.001
	MXF/CAS vs. MEM/CAS	0.56	0.79	0.77	>0.99	0.81	0.98	0.84	0.8	>0.99	0.35	0.02	0.94

Within each antimicrobial, compare enzymes		*S. aureus* in Sa:Ec:Ca	*E. coli* in Sa:Ec:Ca	*C.albicans* in Sa:Ec:Ca	*S. aureus* in Sa:Ca	*C.albicans* in Sa:Ca	*E. coli* in Ec:Ca	*C.albicans* inEc:Ca	*S. aureus* in Sa:Ec	*E. coli* in Sa:Ec	*S. aureus* in Sa	*E. coli* in Ec	*C.albicans* in Ca
No antim	No enzyme vs. Subtilisin A	0.77	0.7	0.69	0.59	0.26	0.85	0.25	0.97	0.9	0.9	0.94	0.61
	No enzyme vs. Ce/De/Di/Ly	>0.99	0.92	0.62	0.78	0.98	0.33	0.84	0.96	0.83	>0.99	0.64	>0.99
	Subtilisin A vs. Ce/De/Di/Ly	0.72	0.91	0.19	0.95	0.18	0.64	0.1	>0.99	0.99	0.86	0.45	0.56
MXF/CAS	No enzyme vs. Subtilisin A	0.42	>0.99	0.15	0.94	0.008	0.76	0.03	0.68	0.75	0.98	0.83	0.01
	No enzyme vs. Ce/De/Di/Ly	0.88	0.9	0.8	0.98	0.15	0.42	0.07	0.86	0.78	0.46	0.05	0.69
	Subtilisin A vs. Ce/De/Di/Ly	0.71	0.87	0.06	0.86	0.32	0.84	0.88	0.94	>0.99	0.58	0.16	0.07
MEM/CAS	No enzyme vs. Subtilisin A	0.82	0.77	0.85	0.89	0.34	0.34	0.02	0.93	0.86	0.41	0.89	0.03
	No enzyme vs. Ce/De/Di/Ly	0.86	0.97	0.73	0.34	0.31	0.81	0.09	0.84	0.7	0.56	0.92	0.14
	Subtilisin A vs. Ce/De/Di/Ly	>0.99	0.9	0.98	0.17	>0.99	0.13	0.68	0.98	0.95	0.96	0.68	0.71

Table S6. MICs (mg/L) of antimicrobials against the strains used in this work.

Strain	Moxifloxacin	Meropenem	Caspofungin
S. aureus ATCC 25923	0.063	0.031	32
S. aureus 5706	0.125	0.125	64
S. aureus 8066	0.063	0.125	64
E. coli ATCC 47076	0.063	0.031	64
E. coli 6081	0.063	0.031	128
E. coli 5701	0.031	0.125	256
C. albicans ATCC 24433	>512	>256	0.125
C. albicans 2522	>512	>512	0.063
C. albicans 7729	>512	>512	0.125

Table S7. One-way ANOVA results from Figure 6. p-value of Tukey's multiple comparisons test. p-values lower than 0.05 are represented in red.

	Subtilisin A MXF/CAS	Subtilisin A MEM/CAS	Ce/De/Di/Ly MXF/CAS	Ce/De/Di/Ly MEM/CAS
ATCC vs. 5706:6081:2522	0.06	0.11	0.4	0.73
ATCC vs. 8066:5701:7729	<0.001	<0.001	0.01	0.02
5706:6081:2522 vs. 8066:5701:7729	0.01	0.01	0.13	0.07
